# Comparison of physiologically based pharmacokinetic modeling platforms for developmental neurotoxicity in vitro to in vivo extrapolation

**DOI:** 10.1093/toxsci/kfaf147

**Published:** 2025-10-27

**Authors:** Anna Kreutz, Xiaoqing Chang, Michael Lawless, Susana Proença, Stephan Schaller, Nicole Kleinstreuer, Helena T Hogberg

**Affiliations:** Inotiv, RTP, Morrisville, NC 27560, United States; Inotiv, RTP, Morrisville, NC 27560, United States; Simulations Plus, Lancaster, CA 42505, United States; ESQlabsGmbH, Saterland 26683, Germany; ESQlabsGmbH, Saterland 26683, Germany; NIH/NIEHS/DTT/NICEATM, RTP, NC 27560, United States; NIH/DPCPSI, Bethesda, MD 20892, United States; NIH/NIEHS/DTT/NICEATM, RTP, NC 27560, United States

**Keywords:** developmental neurotoxicity, in vitro to in vivo extrapolation, physiologically based pharmacokinetic modeling, risk assessment, new approach methods

## Abstract

An extensive battery of 17 in vitro assays has been developed for assessing developmental neurotoxicity (DNT), with the aim of replacing or supplementing traditional in vivo guideline studies for risk assessment, as these mechanistic assays provide advantages over costly, lengthy in vivo studies. However, 1 major challenge in employing in vitro assays is the translation of in vitro bioactive concentrations into in vivo doses that can be compared with human exposures. This study describes an in vitro to in vivo extrapolation (IVIVE) approach to derive human-relevant administered equivalent doses based on chemical partitioning into DNT target organs during the critical period of brain development. We used data from chemicals previously found to elicit bioactivity in a subset (7 of 17) of the in vitro DNT battery assays conducted at the US Environmental Protection Agency. Three physiologically based pharmacokinetic modeling platforms were evaluated for their suitability for this DNT-IVIVE approach. Chemical predictions for administered equivalent doses were compared against in vivo effect levels, where available, and found to be within 3-fold for 78% of chemicals. To provide metrics for risk assessment considerations, administered equivalent doses were compared with predicted human exposures. Overall, this DNT-IVIVE approach was found to be relatively transferable among modeling platforms, albeit with varying limitations and considerations that should be taken into account for specific contexts of use.

With the rising rates of diagnoses of neurodevelopmental disorders over the past several decades, there is increasing demand for testing of chemicals for their potential to induce developmental neurotoxicity (DNT) (Grandjean and Landrigan 2006; Zablotsky et al. 2019; Wang et al. 2021). Traditionally, DNT testing has been performed according to the Organisation for Economic Co-operation and Development (OECD) in vivo DNT Test Guideline 426 (OECD 2007) or Extended One-Generation Reproductive Toxicity Study, DNT Cohort Test Guideline 443 (OECD 2018). However, there are numerous drawbacks to in vivo guideline assays, including costs, time, animal welfare, human relevance, interpretability, and data quality. Only about 200 of the 80,000 chemicals in commerce have been tested in these studies (Luz and Tokar 2018; Wang et al. 2020; Sachana et al. 2021; Crofton and Mundy 2024).

To address these limitations, initial guidance on a battery of 17 in vitro DNT assays (DNT-IVB) was endorsed by OECD (OECD 2023). This DNT-IVB allows for mechanistic insight and assessment of neurodevelopmental key processes using mainly human cells, which in vivo guideline studies cannot provide (Bal-Price et al. 2018; Masjosthusmann et al. 2020). However, to harness the application of in vitro assays for human health risk assessment, in vitro activity concentrations need to be converted into human in vivo doses. In vitro to in vivo extrapolation (IVIVE) allows the translation of an in vitro point-of-departure (POD) into an administered equivalent dose (AED), the external dose required to achieve internal concentrations, typically in plasma, equivalent to concentrations at which in vitro bioactivity was observed. Known neurodevelopmental toxicants have been shown to be active in the DNT-IVB (Druwe et al. 2016; Frank et al. 2017; Harrill et al. 2018; Carstens et al. 2022; Blum et al. 2023), and comparisons have shown this activity to occur at concentrations comparable to or lower than plasma concentrations or lowest-observed-effect levels (LOELs) in vivo in several cases (Shafer et al. 2019; Maass et al. 2023; Bloch et al. 2024; Kreutz et al. 2024). The US Environmental Protection Agency (EPA) used IVIVE based on the DNT-IVB and human steady-state plasma concentrations to support the first waiver of an in vivo guideline DNT study (Dobreniecki et al. 2022). However, plasma concentrations may differ from concentrations at the site of brain development during the critical period of neurodevelopment.

Neurodevelopment consists of a complex series of overlapping events, with a particularly critical period spanning from the second trimester of pregnancy through the first year of life (Rice and Barone 2000; Rock and Patisaul 2018). The fetal brain is separated from the maternal plasma by the fetoplacental barrier and the developing blood–brain barrier. Barrier capacities for both of these change dynamically during neurodevelopment. The fetoplacental barrier is continuously remodeled throughout pregnancy to meet the changing physiological needs of the fetus as it continues to grow. The blood–brain barrier is functional by the 14th gestational week (GW) and continues to mature throughout pregnancy and after birth (Goasdoué et al. 2017; Saili et al. 2017; Ghersi-Egea et al. 2018).

Standard IVIVE analyses use predictive approaches to relate internal concentrations of drugs to external exposure doses. One approach widely used for IVIVE is physiologically based pharmacokinetic (PBPK) modeling, which captures physiological changes, accounts for chemical partitioning between plasma and tissue compartments, and incorporates absorption, distribution, metabolism, and excretion (ADME) processes to estimate tissue-specific concentrations. Literature availability of anatomical and physiological measures during various life stages has enabled the generation of mathematical algorithms describing these changes (Zhang et al. 2017; Abduljalil et al. 2018; Kapraun et al. 2019, 2022). A PBPK model uses a series of differential equations to capture these dynamic processes for healthy adult, child, or pregnant populations. PBPK models can therefore be used to estimate chemical concentrations in tissue compartments at target sites and throughout the critical period of neurodevelopment.

Numerous PBPK modeling platforms exist, with varying degrees of complexity and accessibility (Bouzom et al. 2012). Free, open-source tools are ideal for data sharing and regulatory application. However, compared with publicly available tools, proprietary tools may have advantages such as model complexity, additional population libraries, rich proprietary data sources, and more intuitive interfaces. As predictive tools may provide disparate results, careful consideration must be given to deciding which modeling platform to use, particularly in the realm of risk assessment.

This project broadly examined the impact of different modeling platforms on a DNT-IVIVE approach that was specifically designed to address regulatory needs. We developed a DNT-IVIVE approach using PBPK modeling that considers chemical distribution into DNT-relevant tissue compartments during the critical period of neurodevelopment. This allows for estimation of DNT risk based on in vitro assay data. We applied this DNT-IVIVE approach using 3 PBPK modeling platforms to provide high-level assessments of the utility of these platforms. We utilized a set of chemicals bioactive in 7 of the 17 DNT in vitro assays performed at EPA for which in vitro toxicokinetic data had previously been generated. PBPK modeling was performed at 4 timepoints (prenatal: 15 and 24 GW; postnatal: 2 weeks and 6 months) spanning major critical periods for neurodevelopment. This approach provided human AEDs based on in vitro DNT bioactivity and modeled target tissue maximal concentrations (C_max_) to evaluate potential DNT susceptibility. The utility of this predictive toxicology approach was demonstrated through a comparison of AEDs to in vivo PODs and human biomonitoring data, alongside the derivation of bioactivity exposure ratios (BERs), margin-of-exposure metrics useful in risk assessment prioritization.

## Materials and methods

### Chemical selection

Data from in vitro DNT assays conducted at EPA were obtained in April 2024 from invitroDB version 4.1 (Brown et al. 2023). Data were filtered for high-confidence hits based on a minimum hit call of 0.9 and a maximum of 3 flags. Data were subset for representative samples using the tcplsubsetchid() function in the tcpl R package (Filer et al. 2015). These 7 assays measured a total of 44 endpoints, covering processes including proliferation, apoptosis, synaptogenesis and maturation, cortical neurite outgrowth, human neurite outgrowth, and network formation (EPA 2021) (Table 1 and online supplementary material, Data 1, tab “DNTassays”). Cytotoxic endpoints and the GABA iCell endpoints were excluded. Using the activity concentration at 50% (AC50) as the bioactive concentration, data were filtered for chemicals that produced hits in 3 or more of the 44 endpoints measured collectively by these assays, resulting in a set of 169 “active” chemicals. This chemical set was further narrowed to those for which experimental data for toxicokinetic parameters—in vitro intrinsic hepatic clearance (Cl_int_) and fraction unbound in plasma (f_up_)—were available in the National Institute of Environmental Health Sciences’ Integrated Chemical Environment (ICE; https://ice.ntp.niehs.nih.gov/) v4.1.1 (Wetmore et al. 2012, 2015; Wambaugh et al. 2019; Bell et al. 2020), resulting in a final set of 91 chemicals. The minimum value of f_up_ was set to 0.005.

**Table 1.** DNT in vitro assays used in the DNT-IVIVE approach.

Neurodevelopmental process	Assay	Species	No. of endpoints^a^	No. of active chemicals	No. of chemicals most sensitive
NPC proliferation	hNP1 Prolif	Human	1	11	1
NPC Apoptosis	hNP1 Apop	Human	1	26	3
Neurite outgrowth	hN Initiation^b^	Human	3	27	4
Neurite outgrowth	Cortical Initiation	Rat	3	30	0
Neurite maturation*	Cortical Maturation	Rat	4	39	6
Synaptogenesis*	Cortical Synapto	Rat			
Neural network formation	Cortical MEA	Rat	17	90	78

a Number of endpoints indicates the number of non-viability endpoints measured in the assay.

b hN initiation includes data from both the CDI and hN2 cell lines.

* Neurite maturation and synaptogenesis is a joint assay, so the number of endpoints and chemicals is shared between the two.

NPC, neural progenitor cell.

### PBPK modeling workflow

**Fig. 1.**
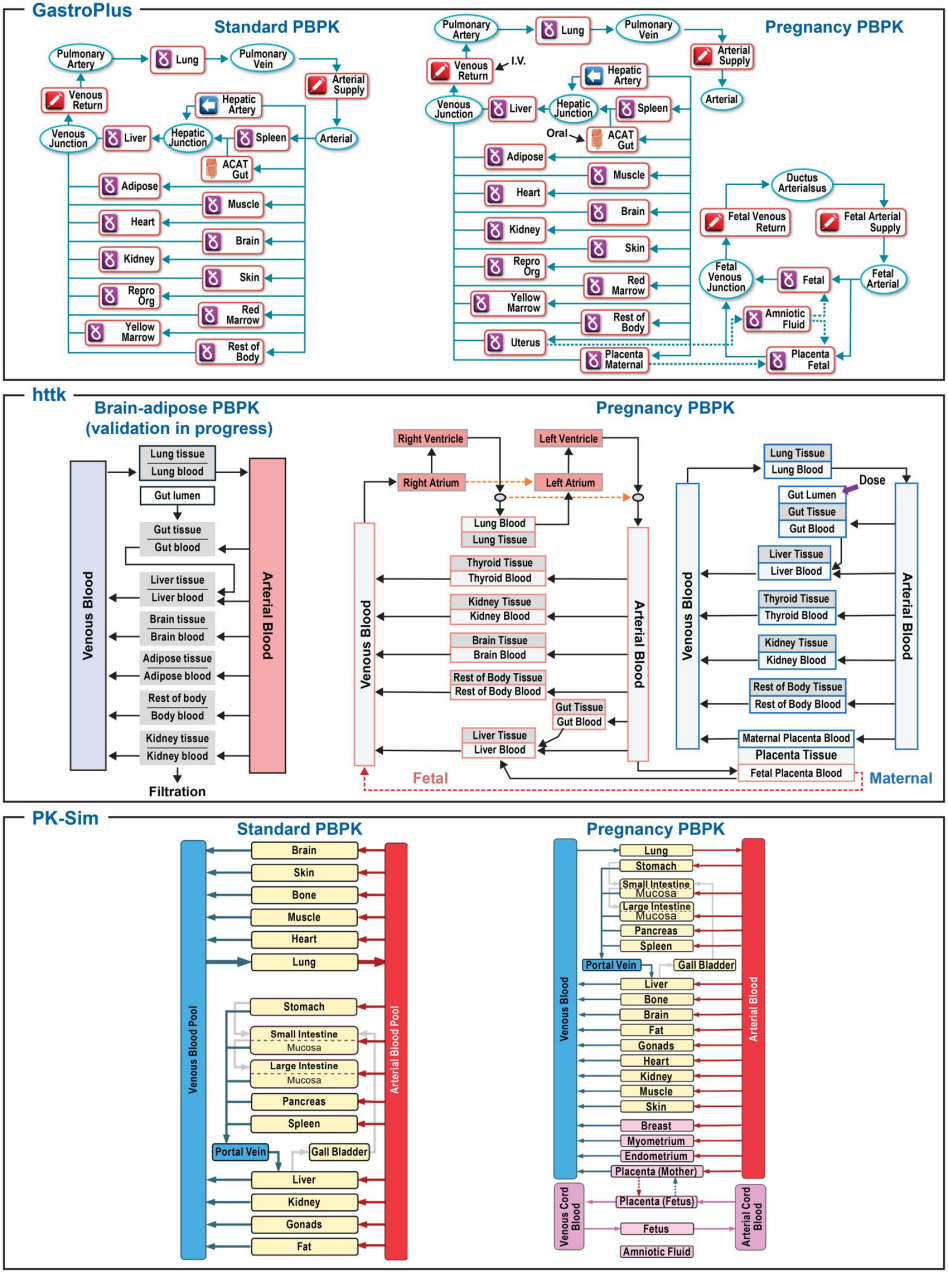
PBPK models. Schematics of pregnancy and standard PBPK models used. ACAT, Advanced Compartmental and Transit model; GI, gastrointestinal; I.V., intravenous; Org, organs.

PBPK modeling was performed in 3 platforms—the commercial software GastroPlus version 9.8.3 (Simulations Plus), the EPA’s open-source R package httk version 2.5.0 (Wambaugh et al. 2024), and PK-Sim version 11.1 from the Open-Systems Pharmacology Suite (PK-Sim 2023; Dallmann et al. 2024). Both the standard and pregnancy PBPK models in each platform were used. As a brain compartment is not yet available in httk, we used a httk brain–adipose model, currently under development by our group based on httk v2.2.2 (Unnikrishnan et al. in prep). For running PK-Sim in batch mode, the developer’s R package (Open Systems Pharmacology 2023) was used, and additional code was developed to facilitate batched running of the PBPK modeling (https://doi.org/10.5061/dryad.6djh9w1fv and https://github.com/esqLABS/pregnancy-neonates-batch-run/tree/master). Schematics of these PBPK models are shown in Fig. 1. Experimental toxicokinetic data—f_up_ and Cl_int_—were provided for all chemicals in each of the platforms. Defaults were used for all remaining parameters, apart from where additional information was needed in PK-Sim.

GastroPlus is a PBPK modeling software traditionally accessed through a graphical user interface (GUI) that is linked with the ADMET Predictor (Simulations Plus) software, which provides predictions of physicochemical and toxicokinetic properties. ADMET Predictor predictions are used by default in GastroPlus to populate chemical properties, including octanol:water partition coefficient (logP), acid dissociation constant (pKa), aqueous solubility, solubility in simulated gastric fluids, and human jejunal permeability. Chemicals were imported from ADMET Predictor as Simplified Molecular Input Line Entry System (SMILES) strings with the provided experimental toxicokinetic data. The fraction unbound in the hepatocyte incubation was set using the method of Austin et al. (2002). By default, tissue-to-plasma partition coefficients (Kp’s) are calculated in GastroPlus using the Lukacova (Rodgers-Single) method (Rodgers et al. 2005; Rodgers and Rowland 2006).

EPA’s httk R package is traditionally run in the R environment. Although a GUI for httk is available in ICE, we did not employ this as it does not allow the degree of parameterization needed for the batched DNT-IVIVE approach applied here (Bell et al. 2020). Physicochemical and toxicokinetic properties used in httk are derived from both experimental data and predictions from the OPEen (Quantitative) Structure–activity/property Relationship App (OPERA) (Mansouri et al. 2019). Although OPERA provides predictions of applicability domain in its standalone GUI as well as ICE, these are not available in httk. An adjustment of the Schmitt method is used by httk to calculate Kp values (Schmitt 2008; Pearce et al. 2017).

PK-Sim is an open-source tool that includes internal quantitative structure–activity relationship (QSAR) models for permeability through the gut and into tissues and Kp values between tissues and plasma and blood and plasma. Similar to GastroPlus, PK-Sim is typically accessed through a GUI. There is no “default” Kp method in PK-Sim, so the method used must be explicitly selected by the user. We used the “PK-Sim standard” partition method, as it was shown to perform the best for high-throughput PBPK modeling in a recent study (Geci et al. 2024). In addition to f_up_ and Cl_int_, PK-Sim requires input values for lipophilicity and solubility, as well as the selection of a Kp prediction method. Lipophilicity predictions were obtained from OPERA. Solubility was set to 1 g/ml to ensure the chemical was fully dissolved.

Three factors were considered in the selection of timepoints for PBPK modeling: Sensitive windows for neurodevelopment, the processes captured by the DNT in vitro assays, and physiological changes in infants. Neurodevelopment begins in the first trimester of pregnancy and continues through the first postnatal year and beyond, with the major sensitive windows for neurodevelopmental disorders occurring throughout pregnancy (Rice and Barone 2000; Semple et al. 2013; Heyer and Meredith 2017; Rock and Patisaul 2018; Wallois et al. 2020). In concordance, the DNT in vitro assays considered here capture processes beginning around the first trimester (e.g. the human neural progenitor cell 1 [hNP1] proliferation assay) and extending beyond birth (synaptogenesis and neural network formation) (Rice and Barone 2000; Frank et al. 2017; Harrill et al. 2018). We did not perform PBPK modeling for the first trimester of pregnancy as (i) most pregnancy PBPK models begin at the second trimester and (ii) due to the lack of a placental barrier and fetal immaturity, maternal plasma is a reasonable surrogate for fetal exposures (GastroPlus 2021; O’Brien and Wang 2023; PK-Sim 2023).

Physiological processes are a particularly important consideration during the first years of life due to the numerous developmental changes occurring during this period, such as in tissue size, protein binding, and hepatic and renal clearance. These shifts begin within hours after birth. Some processes reach near-adult levels of function within the first few months of life, whereas others stabilize within about 6 months to 1 year after birth (Ginsberg et al. 2004; Hines 2013; Cohen Hubal et al. 2014). The overall half-life of chemicals in infants reaches rates similar to adults by 2 to 6 months (Ginsberg et al. 2004). Therefore, to capture the major sensitive periods of neurodevelopment, the window of neurodevelopmental processes measured in the DNT in vitro assays, and variability in physiology over this timeframe, we performed PBPK modeling for the start of the second trimester (15 GW), the start of the third trimester (24 GW), and 2-week (2 w) and 6-month (6 m) postpartum infants.

PBPK modeling was conducted in batch mode in each platform using a pregnancy model at 15 and 24 GW. For modeling of infants, a standard PBPK model was run at 2 w and 6 m of age for each platform; for httk, this was the preliminary brain–adipose model (Unnikrishnan et al. in prep). The individual for simulation was created based on age for GastroPlus and PK-Sim as both platforms account for ontogeny. As httk does not consider ontogeny, age was set in httk by body weight using the GastroPlus default weights for a 2w- and 6 m-old male, which were 3.7 and 8.29 kg, respectively. Chemical exposure was modeled as a single oral bolus of 1 mg/kg body weight and was simulated for a 24-h period. The “immediate release (IR) solution” was used in GastroPlus to simulate an oral dose. As the maximum number of integration steps that GastroPlus can run in batch mode is 500, the integration time interval was divided into 500 steps for all platforms to ensure equivalent granularity across platforms for estimation of C_max_. It is worth acknowledging that this oral exposure does not capture the breadth of chemical exposures infants encounter, particularly lactational transfer or repeat-dose exposure.

Values for C_max_ were derived for DNT-relevant compartments: fetal, fetal venous, and maternal plasma at 15 and 24 GW; brain and plasma at 2w and 6 m; and fetal brain at 15 and 24 GW in httk. To allow for comparison between models, code was written in Python to compute a single effective fetal compartment concentration by volume-averaging the concentrations of the fetal sub compartments (code is available at https://doi.org/10.5061/dryad.6djh9w1fv and https://github.com/esqLABS/pregnancy-neonates-batch-run/tree/master).

PBPK modeling was conducted in batched mode to enable a mid-throughput approach. GastroPlus provides a GUI to allow for batched runs of chemicals, making this easy to implement. For httk, the R code was written to allow for batched modeling using both the pregnancy and brain–adipose models. Several chemicals would not run due to falling outside httk’s applicability domain. Therefore, these chemicals were forced to run using the command, “physchem.exclude = FALSE” or “class.exclude = FALSE.” Although PK-Sim is typically run using a GUI, the GUI does not allow for batched modeling. Therefore, code was written in R to perform batched modeling for PK-Sim. The R code for both httk and PK-Sim is freely available on Dryad and GitHub (https://doi.org/10.5061/dryad.6djh9w1fv and https://github.com/esqLABS/pregnancy-neonates-batch-run/tree/master).

### Reverse dosimetry

IVIVE was performed to derive DNT-specific AEDs—fetus for pregnancy, fetal brain for the httk pregnancy model, and brain for infants. Equation (1) describes calculation of these AEDs using as inputs the AC50 for the lowest in vitro DNT bioactive concentration and the PBPK model-predicted C_max_ for the respective compartment, as shown in Equation (1) (Wetmore et al. 2012):


(1) $$\mathrm{AED}\;(mg/kg/d)=AC50(µM)\times (\frac{1mg/kg/d}{µM\;C_{\max}\;})$$

We used C_max_ in the AED calculations as it is a metric commonly employed for hazard assessment (Haddad et al. 2010; Moser et al. 2015).

### Incorporation of human exposure predictions

Human exposure estimates were derived from Systematic Experimental Evaluation of Methods 2 (SEEM2) predictions available via the EPA’s CompTox Dashboard (Williams et al. 2017) (online supplementary material, Data 2, tab “BERs”). The SEEM2 meta-model incorporates 13 different exposure models and information on human intake rates to predict chemical exposure pathways and intake rates (Ring et al. 2017). BERs allow for doses derived from high-throughput screening hazard data to be compared with exposures for risk assessment prioritization (Webster et al. 2019). From the initial set of 91 chemicals, the 15 chemicals with the lowest AEDs were compared against exposure estimates for the 95th percentile of reproductive-age females to derive a BER using Equation (2):


(2) $$\mathrm{BER}=\frac{\mathrm{AED}\;(mg/kg/d)}{\mathrm{Exposure}\;(mg/kg/d)}$$

The same exposure estimate was used for both the pregnancy and infant timepoints as SEEM predictions are not available for infants; this is further discussed in “Uncertainties” Section. The AED used was the lowest AED derived from each of the 4 ages.

### Curation of in vivo data

In vivo data were gathered from the European Food Safety Authority (EFSA) dataset of in vivo DNT studies of pesticides (Mangas et al. 2025). The dataset was filtered for chemicals assessed in our study, and chemicals with the following annotations were removed to identify DNT-specific effect doses: Generation: parental; effect direction: no effect; endpoint target: body weight, morbidity, vaginal patency, balanopreputial separation. The lowest effect dose for each chemical in the remaining dataset was then used as the in vivo “POD” for comparison to AEDs. These doses were typically the lowest observed adverse effect levels (LOAELs). Further details of the in vivo data curation are provided in Table 2 and online supplementary material, Data 3.

**Table 2.** In vivo DNT PODs from EFSA’s in vivo DNT pesticide database and literature data.

Name	CASRN	Exposure	Endpoint type	POD	AED range^a^	In?
6-Propylthiouracil	51-52-5	Gavage/Intubation; GD6-PND10	FOB; Motor_activity; Startle; Learning_memory; Morphometrics	3.8	3.19 - 516	*
Abamectin	71751-41-2	Gavage/Intubation; GD6-LD22	In_life_Observation	0.4	6.25×10^−4^ - 892	*
Azinphos-methyl	86-50-0	Feed; GD0-LD21	None	NA	3.17 - 299	
Beta-Cyfluthrin	68359-37-5	Feed; GD0-LD21	Pathology_gross	17.8	0.466 - 266	*
Bifenthrin	82657-04-3	Feed; GD0-LD21	FOB	7.2	0.014 - 4.76	**
Carbaryl	63-25-2	Gavage/Intubation; GD6-LD10	Morphometrics	10	0.469 - 33.2	*
Carbofuran	1563-66-2	Feed; GD6-LD10	Learning_memory; Pathology_gross	4.95	1.06 - 10.6	*
Chlorpyrifos	2921-88-2	Gavage/Intubation; GD6-LD11	Morphometrics	1	0.580 - 102	*
Coumaphos	56-72-4	Feed; GD0-LD21	Cholinesterase; Morphometrics	2.44	5.84 - 228	**
Diazinon	28647-38-3	Feed; GD0-LD21	Sexual_landmark; Motor_activity; Pathology_gross; Cholinesterase	24.2	5.71 - 127	*
Dimethoate	60-51-5	Gavage/Intubation; P11-P21	Motor_activity	0.5	9.88 - 1110	
Emamectin Benzoate	155569-91-8	Gavage/Intubation; GD6-LD20	Motor_activity	0.6	0.00533 - 2630	*
Endosulfan	115-29-7	Feed; GD6-LD21	In_life_Observation	29.8	0.378 - 36.1	*
Ethoprop	13194-48-4	Feed; GD6-LD21	Motor_activity	0.7	1.75 - 32.9	*
Fenpropathrin	39515-41-8	Feed; GD6-LD21	In_life_Observation; Motor_activity; Startle; Pathology_gross	19	0.0725 - 24.5	*
Fipronil	120068-37-3	Feed; GD6-LD10	Developmental_landmarks; Startle; Learning_memory; Pathology_gross	15	0.660 - 49.6	*
Flufenacet	142459-58-3	Feed; GD6-P10	Startle; Morphometrics	1.7	1.14 - 99.7	*
Haloperidol	52-86-8	IP; E6-16	Learning_and_memory; Morphometrics	2.5	0.0514 - 87.5	*
Heptachlor	76-44-8	Oral; GD12-P7	In_life_Observation	0.03	0.0353 - 26.3	
Indoxacarb	173584-44-6	Gavage/Intubation; P11-P21	In_life_Observation	3	7.87 - 313	**
Malathion	121-75-5	Gavage/Intubation; P11-P21	In_life_Observation; FOB	150	28.1 - 1.44×10^4^	*
Methotrexate	59-05-2	IP; P1-5	Morphometrics	0.05	0.0139 - 7.24×10^7^	*
Methyl Parathion	298-00-0	Gavage/Intubation; LD11-LD21	In_life_Observation	0.6	6.82 - 47.6	
Molinate	2212-67-1	Feed; GD7-LD11	Startle	6.9	0.290 - 3.87	**
Phorate	298-02-2	Gavage/Intubation; P11-P21	Learning_memory	0.1	7.66 - 68.7	
Rotenone	83-79-4	IP; P5-P11	Hyperlocomotion; Social interaction; Contextual memory	0.1	0.00327 - 0.188	*
S-Bioallethrin	28434-00-6	Oral; P10-P16	Morphometrics; Motor_activity	0.7	0.630 - 27.4	*
Tebuconazole^b^	107534-96-3	Feed; GD14-P42	Morphometrics	6	0.180 - 10.0	*
Terbufos	13071-79-9	Gavage/Intubation; P11-P21	Motor_activity; Morphometrics	0.08	2.88 - 84.1	
Triallate	2303-17-5	Gavage/Intubation; GD6-LD20	In_life_Observation; Motor_activity; Learning_memory	60	1.91 - 27.7	
Tribufos	78-48-8	Feed; GD0-P21	Sexual_landmark; Developmental_landmarks; Motor_activity; Startle; Pathology_gross	16.4	4.37 - 93.1	*
Trichlorfon	52-68-6	Feed; GD0-LD21	Startle	13.4	7.05 - 5370	*

Where CASRNs were not provided, the CASRN for the DNT in vitro assay was listed. PODs and AEDs in mg/kg/d. "In?" indicates chemicals for which the POD was within the range of predicted AEDs (*) and chemicals that were within 3-fold (**). Additional information is available in online supplementary material, Data 3.

E, embryonic day; FOB, functional observation battery; GD, gestational day; IP, intraperitoneal; LD, lactational day; P, postnatal day.

a AEDs were derived from a single oral dose.

b For tebuconazole, PODs were available from both the literature and pesticide database, so the more conservative of these, the literature value, is listed.

Additional in vivo data were obtained via a literature search conducted using PubMed and ToxRefDB (Feshuk et al. 2023) for in vivo mammalian DNT studies that provided exposure doses and corresponding anatomical or behavioral changes. Changes in gene expression were not included as a DNT endpoint. The literature search provided relevant in vivo data for 9 chemicals, 3 of which were also included in the EFSA dataset (online supplementary material, Data 3, tab “Literature”). Again, the lowest doses that produced DNT-relevant effects are all referred to here as PODs. To further help benchmark PBPK modeling results, we also obtained data on human fetal cord blood to maternal plasma concentration ratios for 6 chemicals in our test set (Aylward et al. 2014). These were compared against predicted ratios of fetal venous to maternal plasma concentrations at the end of the simulation (Table 3).

**Table 3.** Comparison of model predictions to available human in vivo data.

	Human fetal cord blood: maternal plasma			
	In vivo (mean, min to max)^a^	GastroPlus^b^	httk^b^	PK-Sim^b^
Aldrin	0.6915; *N* = 1	0.941	0.821	0.953
Dieldrin	0.735 (0.708 - 0.762); *N* = 2	0.899	0.599	0.974
Heptachlor epoxide	0.803 (0.365 - 1.241); *N* = 2	0.909*	0.820*	0.978*
Dichlorodiphenyldichloroethane	0.764 (0.109 - 1.214); *N* = 5	0.943*	0.638*	0.961*
Dichlorodiphenyldichloroethylene	0.736 (0.251 - 1.863); *N* = 16	0.497*	0.910*	0.945*
Dichlorodiphenyltrichloroethane	0.640 (0.242 - 1.208); *N* = 10	0.914*	0.827*	0.962*

a In vivo human cord blood samples were taken at birth.

b Ratios from the end of the 24-h PBPK modeling period. Shading indicates PBPK modeling predictions that were within range of in vivo values.

* Within the range of in vivo.

### Sensitivity analysis

To begin to address key parameters in the determination of fetal and brain C_max_ and potential drivers of differences between modeling platforms, we conducted a parameter sensitivity analysis. This was performed in GastroPlus due to our familiarity with their internal tool for parameter sensitivity analysis that includes predefined parameter distributions. Using these default distributions, parameters known to impact chemical internal kinetics were tested individually for their impact on C_max_ (Blaauboer 2010; Wambaugh et al. 2015; Wambaugh et al. 2018). These included Cl_int_, f_up_, blood to plasma ratio, lipophilicity distribution coefficient (logD), effective permeability (P_eff_), reference solubility, and either the fetal or brain tissue partition coefficient—Kp_fetal_ or Kp_brain_—for 24 GW and 6 m, respectively. Although PBPK modeling was performed using logP to provide consistency across models, logD was used for the sensitivity analysis, as logP is not an option in the GastroPlus GUI. Sensitivity analyses were conducted on a set of 5 chemicals that represent a range of toxicokinetic and physicochemical properties. Normalized sensitivity coefficients (NSCs) (Equation 3) were calculated for each parameter to provide an estimate of the relative impact of that parameter on predicted fetal concentrations at 24 GW and brain concentrations at 6 m.


(3) $$\begin{array}{l} \%C=\frac{\left(C3-C2\right)}{C1}\\ \%P=\frac{\left(P3-P2\right)}{P1}\\ NSC=\frac{\%C}{\%P} \end{array}$$

In Equation (3), C (concentration) is the output value and P is the parameter that is adjusted. The numerals 1, 2, and 3 represent the mean, minimum, and maximum values of the distribution, respectively. These are summarized in Table 4.

**Table 4.** Parameter sensitivity analysis in GastroPlus.

Name		Chlorpyrifos	Fipronil	Bifenthrin	Pyraclostrobin	Triamcinolone
Chemical properties		Neutral, low Cl_int_ and f_up_, logP = 4.96	Neutral, Cl_int_ = 0, low f_up_, logP = 4	Neutral, low f_up_, Cl_int_ = 0, logP = 6.19	MPB, low f_up_, high Cl_int_, logP = 3.99	MPA, high f_up_, low Cl_int_, logP = 1.16
**24 GW**	Liver Cl_int_	−0.001	−0.010	−0.001	−0.036	−0.143
	f_up_	0.072	0.173	0.001	**0.309**	−0.055
	logD	**0.501**	**0.467**	**0.420**	**1.150**	−0.189
	RBP	−0.299	−**0.416**	−**0.421**	−**0.372**	0.076
	Kp_fetal_	**0.671**	**0.753**	**0.813**	**0.630**	**0.865**
	P_eff_	0.209	0.287	0.288	0.151	**0.405**
	Ref Sol	0.035	0.047	0	0.037	0.007
**6 M**	Liver Cl_int_	−0.021	−0.043	0	−0.008	−**0.539**
	f_up_	0.057	0.139	0	0.230	−0.135
	logD	**0.642**	0.150	**0.438**	**2.033**	**0.306**
	RBP	−0.227	−0.296	−0.294	−0.230	0.109
	Kp_brain_	**0.536**	**0.606**	**0.618**	**0.754**	**0.899**
	P_eff_	0.147	0.099	0.203	0.121	**0.563**
	Ref Sol	0.047	0.039	0.003	0.031	0.022

Boldface indicates values with an absolute value of 0.3 or more; underlined exceed an absolute threshold of 0.5. Values <0.001 indicated as 0.

Cl_int_, intrinsic hepatic clearance; f_up_, fraction unbound in plasma; Kp, tissue partition coefficient; logD, lipophilicity distribution coefficient; logP, octanol:water partition coefficient; MPA, monoprotic acid; MPB, monoprotic base; P_eff_, effective permeability; RBP, blood to plasma ratio; Ref Sol, reference solubility.

## Results

### DNT-IVIVE results

#### Distribution of PBPK modeling results across platforms

The “C_max_ Distribution” table (online supplementary material, Document 1, Table 1) shows the distribution of C_max_ values for each age, compartment, and model. Eight chemicals could not be run by default for the httk pregnancy model, as they were indicated as falling outside the applicability domain; these included aldrin, bisphenol AF, heptachlor, mirex, N-ethylperfluorooctane sulfonamide, perfluorooctanesulfonamide, p,p-DDE, retinoic acid, and tri-allate. Predictions for these chemicals were obtained using the command, “physchem.exclude=FALSE” or “class.exclude=FALSE.” As a result, httk predictions for these chemicals should be considered with caution. As the predictions for these chemicals did not show a distinct pattern of distribution, they were kept in the test set.

**Fig. 2.**
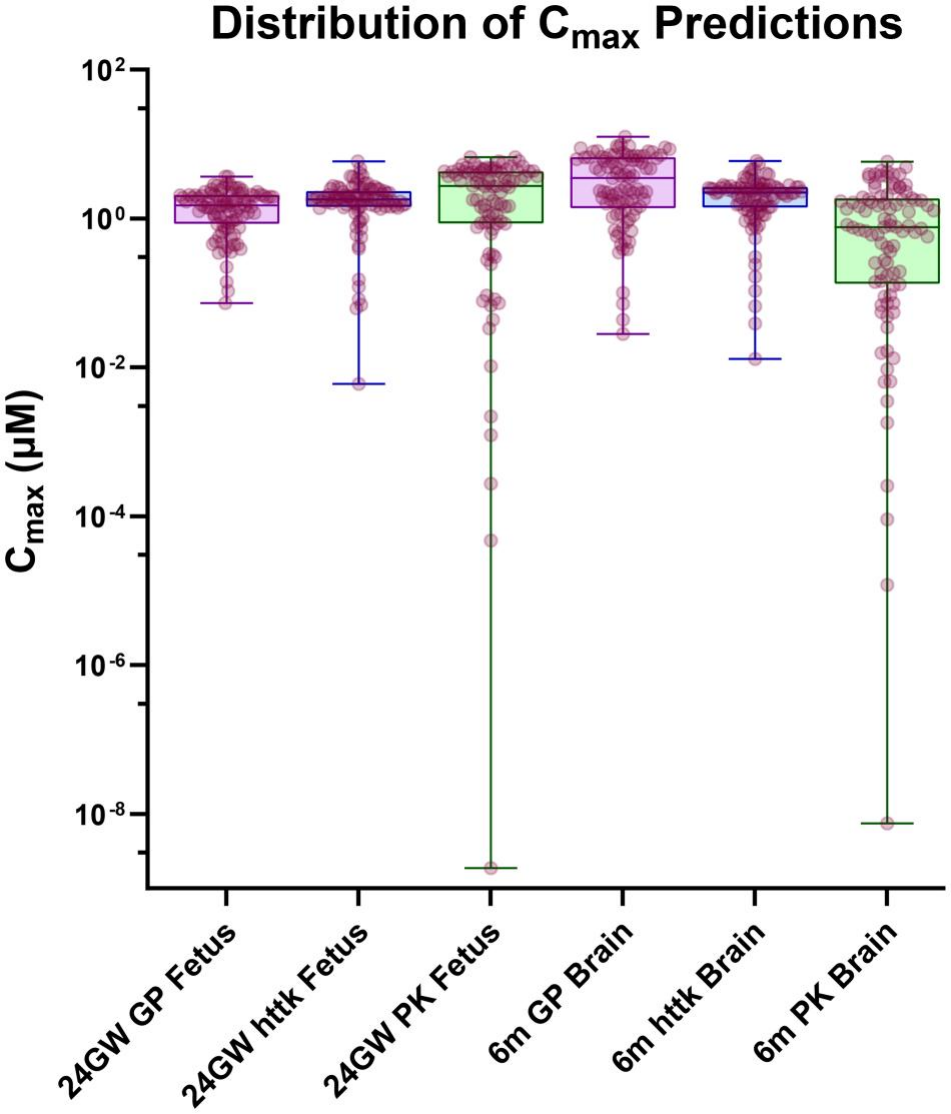
Distribution of C_max_ predictions of the 91 chemicals in the test set for fetus at 24 GW and brain at 6 m from GastroPlus (GP; purple), httk (httk; blue), and PK-Sim (PK; green).

Predictions were relatively concordant among the platforms when simply considering the overall distribution of C_max_ values (Fig. 2). The major difference among the platforms seemed to be that PK-Sim predicted far lower minimum C_max_ values for some compounds across all compartments, particularly in fetus and brain, as compared with GastroPlus and httk. GastroPlus generally predicted higher values of brain C_max_, on average, than httk and PK-Sim. Comparing life stages showed similar ranges of C_max_ for fetus at 15 and 24 GW, and brain at 2 w and 6 m (online supplementary material, Data 2, tab “CmaxComparisons).” This held true for most chemicals on an individual chemical level, with all chemicals falling within 5-fold of one another between 15 and 24 GW for GastroPlus and httk, and between 2 w and 6 m for GastroPlus and PK-Sim. For 23 chemicals, the C_max_ values predicted by PK-Sim at 15 GW differed by more than 5-fold but less than 10-fold from C_max_ values predicted at 24 GW for PK-Sim. The C_max_ values predicted by httk at 2 w differed by more than 10-fold from C_max_ values predicted at 6 m of age for 8 chemicals.

To simplify this analysis, subsequent assessments focused on the 24 GW and 6 m timepoints, as more data exists on physiology at these life stages. Greater differences were observed between modeling platforms for the predicted C_max_ at 6 m as compared with the C_max_ predicted at 24 GW, but for both timepoints, some chemicals differed by more than 100-fold. Differences of such magnitude were exclusively observed in comparisons between C_max_ values from PK-Sim and those from either of the other 2 platforms. These included predictions for abamectin, cytarabine hydrochloride, emamectin benzoate, methotrexate, and pravastatin sodium at 24 GW, and for abamectin, auramine hydrochloride, colchicine, cytarabine hydrochloride, methotrexate, pravastatin sodium, reserpine, and triamcinolone at 6 m. For comparison, the average fold difference between C_max_ predictions from GastroPlus and httk was approximately 2-fold across all chemicals at both 24 GW and 6 m.

To better understand the importance of DNT-specific tissue metrics, e.g. fetus and brain, as compared with general plasma concentrations, DNT tissue C_max_ values were compared against plasma C_max_ values (Table 5). The average ratio of predicted fetal tissue C_max_ values to predicted maternal plasma C_max_ values exceeded 1 for 54% of chemicals in GastroPlus, 80% in httk, and 69% in PK-Sim. For httk, the only model that predicts fetal tissue-specific concentrations, the average ratio of fetal brain to maternal plasma C_max_ also exceeded 1. However, fetal brain C_max_ values were somewhat lower than the lumped fetal tissue C_max_ value for the majority of chemicals, with the ratio never exceeding 1.5 for the few chemicals for which fetal brain C_max_ exceeded fetal C_max_. At 6 m, the ratio of infant brain to plasma C_max_ exceeded 1 for 77% of chemicals in GastroPlus, 54% in httk, and 59% in PK-Sim. This suggests that some of these chemicals may preferentially partition into the fetal and brain compartments as compared with plasma, and thus that DNT target tissue metrics may be more conservative than plasma concentrations. In addition, lumped fetal concentrations, rather than fetal brain-specific concentrations, were generally sufficiently conservative for estimations of chemical concentrations in the developing fetal brain. This suggests that the use of overall fetal rather than fetal brain-specific concentrations would be sufficiently conservative, in addition to simplifying the collection of benchmarking data.

**Table 5.** Relative C_max_ distributions.

	Chemical compartmental partitioning					
	24 GW C_max_ fetus/plasma			6 m C_max_ brain/plasma		
	GastroPlus	httk	PK-Sim	GastroPlus	httk	PK-Sim
Med	1.099	2.059	5.366	2.809	1.098	1.540
Min	0.053	0.262	3.66×10^−5^	0.055	0.019	3.03×10^−4^
Max	1.855	25.538	2700	4.623	14.095	2770
Proportion exceeding 1	54%	80%	69%	77%	54%	59%

Ratios of C_max_ for the fetal tissue compartment to maternal plasma C_max_ at 24 GW and infant brain C_max_ to infant plasma C_max_ at 6 m of age. *N* = 91.

#### Parameter sensitivity analysis

Parameters known to impact chemical internal kinetics were assessed for their impact on fetal and brain C_max_. This was done in GastroPlus due to our familiarity with their internal parameter sensitivity analysis module (Blaauboer 2010; Wambaugh et al. 2015, 2018; Geci et al. 2024). NSCs were calculated to provide an estimate of the relative impact of each parameter on fetal concentrations at 24 GW and brain concentrations at 6 m (Table 4). Five physicochemically and toxicokinetically diverse chemicals were assessed here. Lipophilicity and Kp’s had the largest NSCs, demonstrating their importance in dictating fetus and brain C_max_. P_eff_ also had a relatively higher NSC for triamcinolone, suggesting this parameter might be important in determining distribution for some chemicals.

#### DNT-IVIVE-derived AEDs

**Fig. 3.**
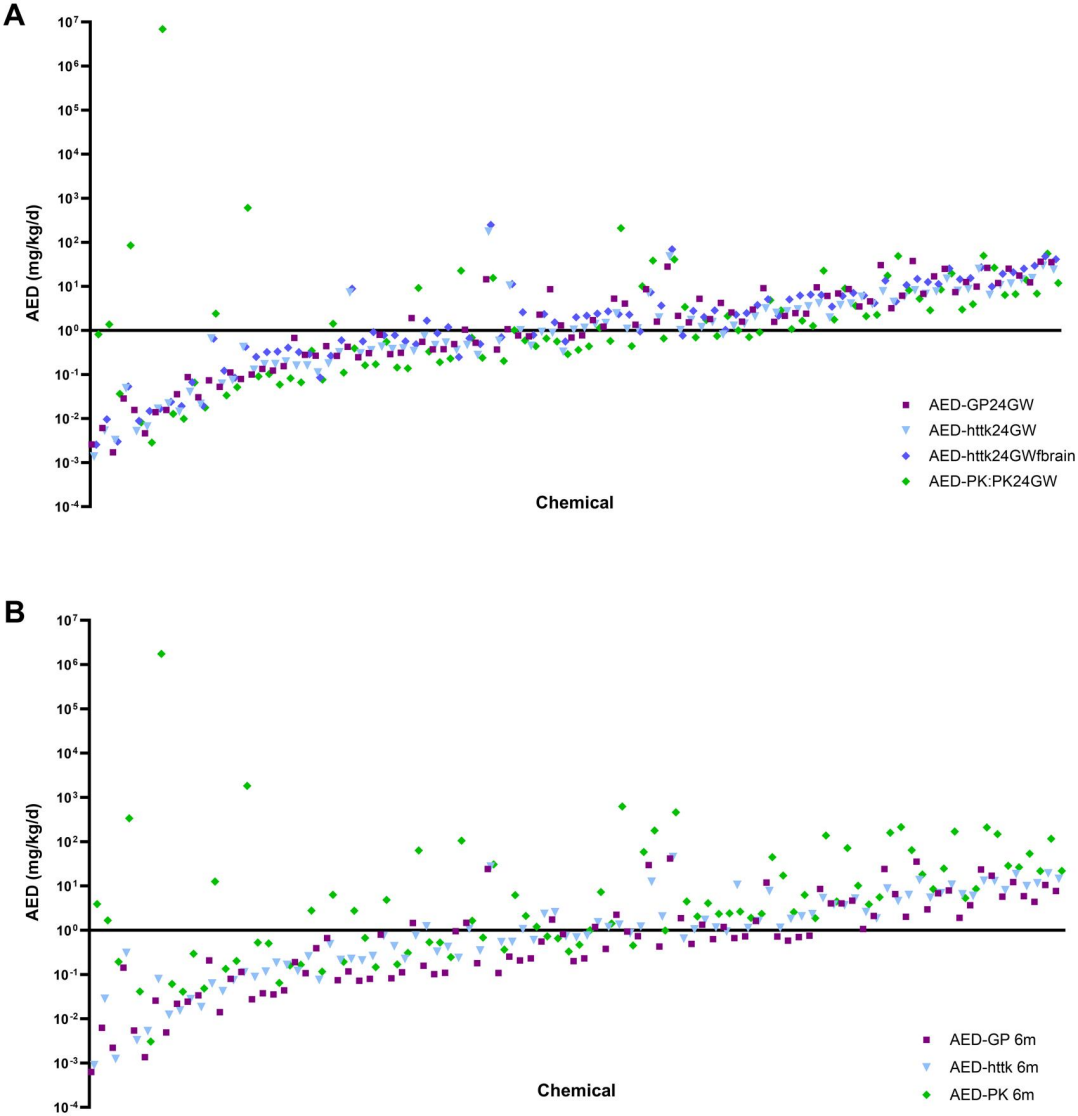
DNT-IVIVE derived AEDs for 24 GW (A) and 6 m (B) based on a single oral exposure. AEDs are ordered by chemical along the *x*-axis from lowest to highest minimum bioactive concentration. Upper graph shows AEDs at 24 GW based on fetal C_max_ for all platforms and fetal brain for httk (httk24GWfbrain). AEDs at 6 m are based on brain C_max_.

To translate bioactivity in DNT in vitro assays into human AEDs, IVIVE was performed using as input the AC50 for the lowest in vitro DNT bioactive concentration and the predicted C_max_ for the DNT-relevant compartments, fetus and brain. The distribution of AEDs for each of the chemicals across the different platforms is plotted in Fig. 3 for the 24 GW and 6 m timepoints. The graph showing AEDs for 24 GW compares fetal brain concentrations derived from httk to overall fetal concentrations from all 3 models, as fetal brain concentrations were only available from httk. Greater concordance among platforms was seen at 24 GW than at 6 m. PK-Sim produced notably higher AEDs for a number of chemicals at both life stages. This difference is further addressed in the “Comparison of modeling platforms” Section.

#### Consideration of human exposure

**Fig. 4.**
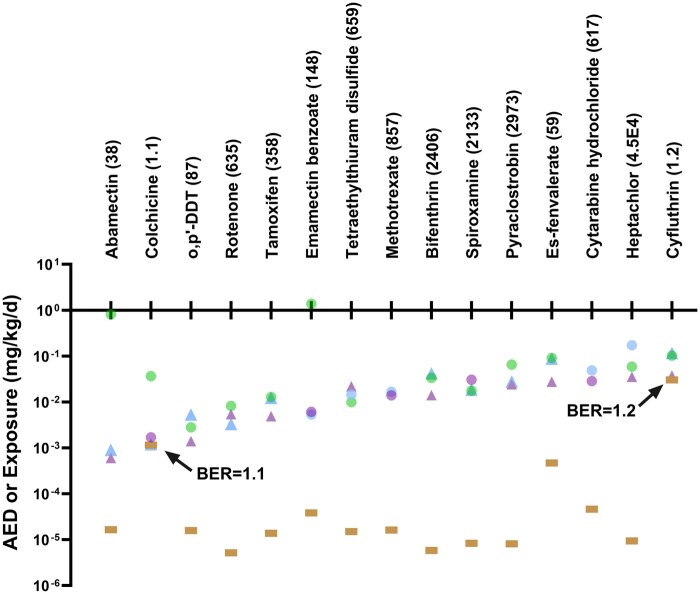
Comparison of IVIVE-predicted AEDs to SEEM2 exposure estimates for reproductive-aged females to demonstrate BERs. Chemicals with the 15 lowest AEDs are plotted for each of the platforms. The BER for each chemical appears in parentheses after the chemical name. The BERs for the 2 chemicals with the lowest BERs are indicated. Colored circles and triangles represent AEDs for 24 GW and 6 m, respectively (GastroPlus: purple, httk: blue, PK-Sim: green). Brown bars are SEEM2 exposure estimates.

Human exposure estimates for reproductive-age females (95th percentile predictions) were gathered from SEEM2 through the CompTox Chemicals Dashboard v2.4.1 for both chemicals with the lowest AEDs and chemicals with in vivo data (Williams et al. 2017). It should be acknowledged that this is a simplified exposure prediction, as SEEM2 does not include exposure predictions for infants. We compared SEEM2 exposure predictions to the corresponding AED for each chemical to derive BERs, a ratio of the AED to the exposure that provides a metric that could be employed in risk assessment and prioritization considerations. To be conservative, the AED used was the lower of either the 24 GW fetal or 6 m brain C_max_. A lower BER suggests a higher probability of public exposure to the chemical at a level sufficient to elicit bioactivity. The 2 chemicals with the lowest BERs were colchicine and cyfluthrin, with BERs of 1.1 and 1.2, respectively (Fig. 4).

### Evaluation of this DNT-IVIVE approach: benchmarking against in vivo data

Benchmarking this, predictive DNT-IVIVE approach is challenging because there are limited relevant in vivo data for chemical concentrations in the brain or fetus, especially for humans and environmental chemicals. However, we were able to identify some in vivo data that could be used for this purpose, specifically in vivo POD data from mammalian studies and human fetal cord blood to maternal plasma concentration ratios.

#### In vivo PODs for DNT

**Fig. 5.**
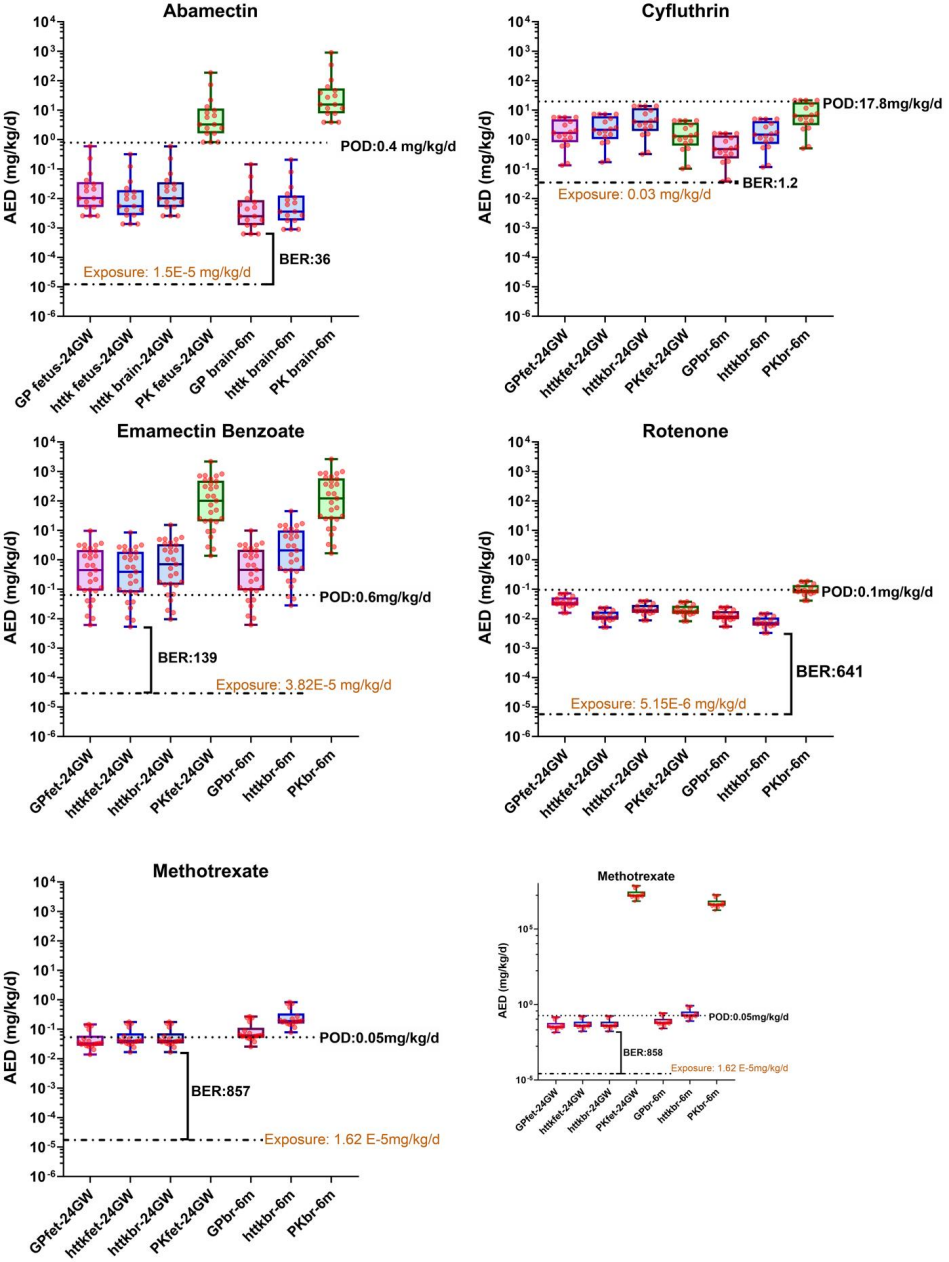
Comparison of in vitro DNT-IVIVE-derived AEDs against curated in vivo DNT PODs for chemicals with a BER below 1000. AEDs for each of the bioactive endpoints are plotted in red for the 3 platforms at 24 GW and 6 m; 95th percentile exposure estimates for reproductive-age females are indicated to provide BERs. For comparative purposes, the *y*-axis is scaled to the same degree for all chemicals. A subset plot is provided for methotrexate to capture the particularly high AEDs in PK-Sim.

In vivo PODs for chemicals assessed here were gathered from the EFSA DNT dataset (Mangas et al. 2025) and a literature review of in vivo DNT studies. Relevant in vivo DNT POD data were identified for 31 chemicals. The majority of PODs came from regulatory LOAEL’s and are further described in the “Curation of in vivo data” Section, Table 2, and online supplementary material, Data 3. Endpoints classified as body weight or sexual landmarks were removed. Boxplots were created to visualize the concordance of AEDs for all in vitro DNT bioactive endpoints from the 3 modeling platforms with in vivo PODs at the 24 GW and 6 m timepoints (Fig. 5 and online supplementary material, Document 1, Fig. 1). The boxplot comparison shows that the majority of chemicals had in vivo PODs that fell within the range of in vitro-derived AEDs (66%; 78% within 3×), showing the concordance of in vitro-derived AEDs and in vivo PODs. Similar concordances were seen across the 3 platforms. In addition, for most chemicals (72%), the lowest AEDs were lower than in vivo PODs. One of these chemicals, azinphos-methyl, had no demonstrated DNT effects in vivo. This indicates that in vitro-derived AEDs might provide more conservative estimates of DNT risk than in vivo tests. PK-Sim tended to give higher AEDs for some compounds, such as abamectin, emamectin benzoate, and methotrexate, a finding that we explore further in “Comparison of modeling platforms” and the “Discussion” Sections. The Fig. 5 graphs also show exposure estimates and corresponding BERs for chemicals for which the BER was less than 1000.

#### Human in vivo data

Human in vivo data were found for 6 of the test set chemicals for the ratio of measured human fetal cord blood to maternal plasma concentrations (Aylward et al. 2014). These were compared against ratios of fetal venous to maternal plasma C_max_ predictions at the end of the 24-h PBPK modeling period, because this was the timepoint from our modeling that would best correlate with steady-state levels in human fetal cord blood samples collected at birth. Overall, predicted ratios fell in the range of in vivo measured values for most chemicals across the 3 platforms (Table 3). The 2 chemicals for which predictions did not fall within the range of in vivo values were chemicals for which only 1 or 2 in vivo datapoints were available. These data support the concordance of PBPK-derived concentrations with in vivo data.

### Comparison of modeling platforms

To better understand drivers’ underlying differences in model outputs, we compared model structures and parameters that influence chemical distribution as determined in the parameter sensitivity analysis (“Parameter sensitivity analysis” Section). We focused these comparisons on the physicochemical properties and parameter values for the 4 chemicals for which C_max_ values differed by more than 1,000-fold between PK-Sim and the other models. These chemicals—abamectin, cytarabine hydrochloride, methotrexate, and pravastatin hydrochloride—were those that showed the lowest C_max_ in PK-Sim at both 24 GW and 6 m (Fig. 2).

#### Structure and physiology

Both the number and connections of compartments can influence PBPK predictions. Each model in this project is structured somewhat differently, as seen in Fig. 1. GastroPlus has 15 compartments in their standard PBPK model and 22 in the pregnancy model, httk has 10 compartments in the brain–adipose model and 20 in the pregnancy model, and PK-Sim has 19 compartments in the standard model and 28 in the pregnancy model. The parameters used for blood flow and volume for the compartments assessed are similar across the 3 platforms, and kidney clearance is based on glomerular filtration rate for each.

One major consideration in model structure is how tissues are limited, which can be either by blood perfusion and/or cellular permeability. By default, the GastroPlus and httk pregnancy models are perfusion-limited, whereas the brain in the httk brain–adipose model and both the brain and placenta in PK-Sim are permeability-limited. In httk, brain permeability is limited using a P_eff_ value to mimic the blood–brain barrier based on in vitro data from parallel artificial membrane permeability assay (PAMPA) experiments (Wang et al. 2022). PK-Sim uses a much more complex series of partitioning steps into the interstitial fluid and endothelial cells, with values of endothelium permeability very high across organs except for the brain and placenta to mimic these barriers. The difference between the perfusion- and permeability-limited models fits with the lower predicted C_max_ seen in the permeability-limited tissues—fetus in PK-Sim and brain in httk and PK-Sim (Fig. 2)—though C_max_ values for the brain in httk were only slightly lower than for GastroPlus. It is worth noting that barriers can also be made permeability-limited in GastroPlus. However, this option was not assessed here, as it is not the default setting.

#### Lipophilicity

**Fig. 6.**
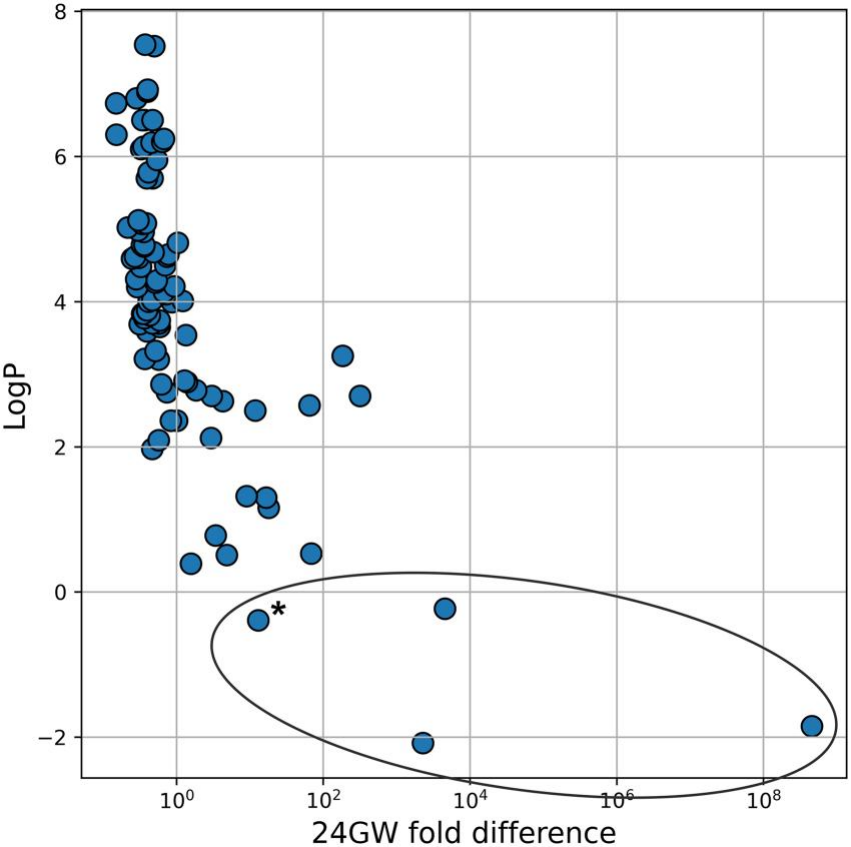
Distribution of chemical test set comparing lipophilicity (logP) to the fold difference in fetal predicted C_max_ between PK-Sim and GastroPlus at 24 GW. The 4 chemicals with the lowest logP values are circled. *6-propyl uracil is flagged as it does not show the extent of difference in C_max_ as the other chemicals with low logP values.

Lipophilicity, expressed as logP, was the most important contributor to chemical distribution into the fetal and brain compartments based on the parameter sensitivity analysis. Figure 6 visualizes the relationship between the logP values of the test set chemicals and the average fold differences of the 24 GW fetal C_max_ predictions from PK-Sim as compared with GastroPlus. The 3 chemicals that differed the most between the platforms were among the chemicals with the lowest logP values, suggesting that lipophilicity may help to explain the difference in predictions from PK-Sim. However, 6-propyl-2-thiouracil, with the third-lowest logP, had a PK-Sim predicted C_max_ that only differed 12-fold from the other platforms, suggesting other factors must also contribute to differences among platforms. It should be noted that while the PBPK modeling was performed using logP values to provide consistency across models, the parameter sensitivity analysis was conducted using logD, as logP was not an option in the sensitivity analysis GUI in GastroPlus.

#### Tissue partition coefficient calculation and comparison

**Fig. 7.**
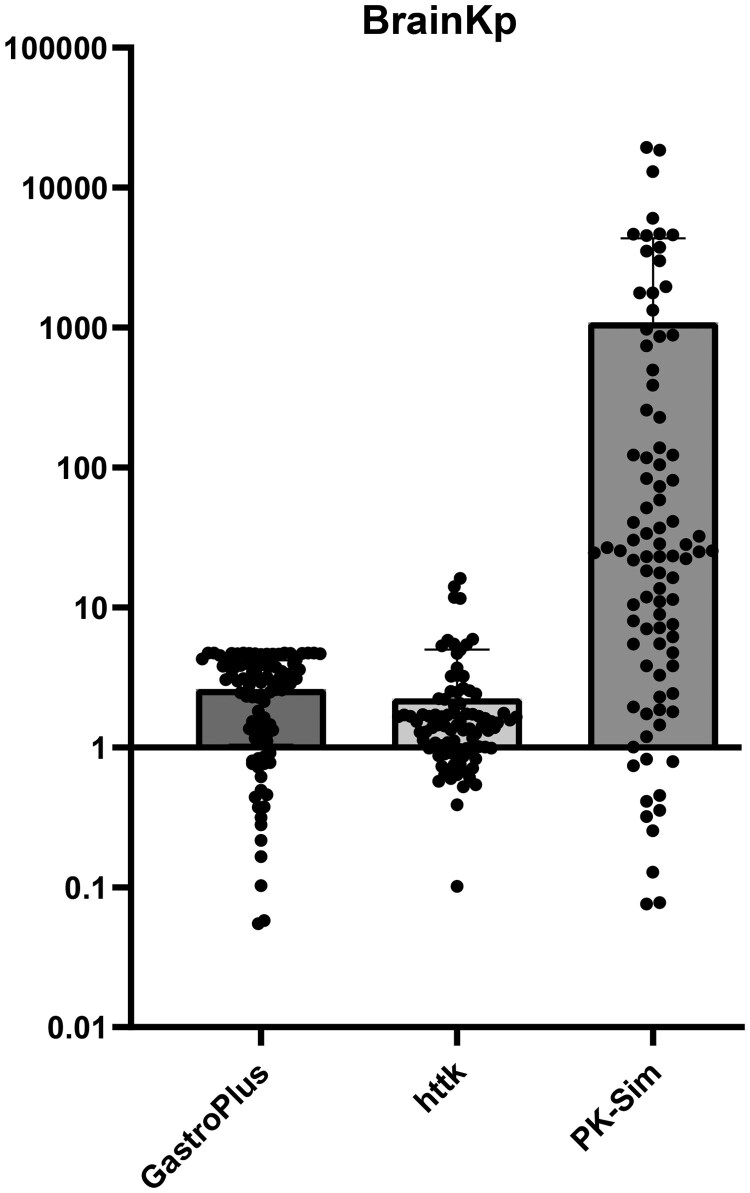
Comparison of Kp values. Brain Kp values for the 6 m PBPK models for GastroPlus, httk, and PK-Sim.

Lipophilicity feeds into the equations for calculating Kp values, and the parameter sensitivity analysis showed Kp values to be another major driver of chemical distribution. As seen in Fig. 7, there is a much broader distribution of brain Kp values for PK-Sim as compared with GastroPlus and httk.

To better understand why Kp values differed substantially for PK-Sim as compared with GastroPlus and httk, the underlying equations for the partitioning methods used by each platform were assessed. By default, GastroPlus uses the Lucakova (Rodgers-single) method, and httk uses the Schmitt method (Rodgers et al. 2005; Rodgers and Rowland 2006; Schmitt 2008). PK-Sim does not provide a default option, so here the internal PK-Sim partitioning method “PK-Sim standard” was selected. Upon further evaluation, we noted 2 major differences in how Kp values are calculated in PK-Sim as compared with the other platforms. One difference is that PK-Sim calculates Kp based on the ratio of intracellular tissue-to-plasma concentration, whereas GastroPlus and httk calculate Kp based on the overall tissue concentration. A cursory assessment of this difference indicated it was unlikely to have a major impact on modeling. Another major difference noted among the models was that GastroPlus and httk apply an automatic f_up_ adjustment that considers lipid binding in calculating Kp values, whereas PK-Sim does not (Lucakova et al. 2008; Pearce et al. 2017). Although the adjustment factor equations differ somewhat between GastroPlus and httk, both models produce similarly adjusted f_up_ values. The lack of a f_up_ adjustment factor might explain the relatively higher Kp values in PK-Sim as compared with GastroPlus and httk.

#### Absorption

Absorption is modeled in GastroPlus and PK-Sim using predictions of permeability, expressed as P_eff_ in GastroPlus and intestinal permeability (P_int_) in PK-Sim. P_eff_ had the third-largest NSC in the parameter sensitivity analysis, suggesting that permeability is an additional important contributor to the levels of C_max_ achieved in brain and fetal compartments. P_eff_ is an experimentally determined measure that can be related to an absorption rate coefficient. PK-Sim calculates P_int_ based on the membrane affinity (MA), which, in principle, is the lipophilicity value used in the model (Equation 4):


(4) $$P_{\mathrm{int}}({MW}_{\mathrm{eff}},MA)=265.796\;\times \;{MW}_{\mathrm{eff}}^{-4.49968}\times MA(\frac{cm}{s})$$

**Fig. 8.**
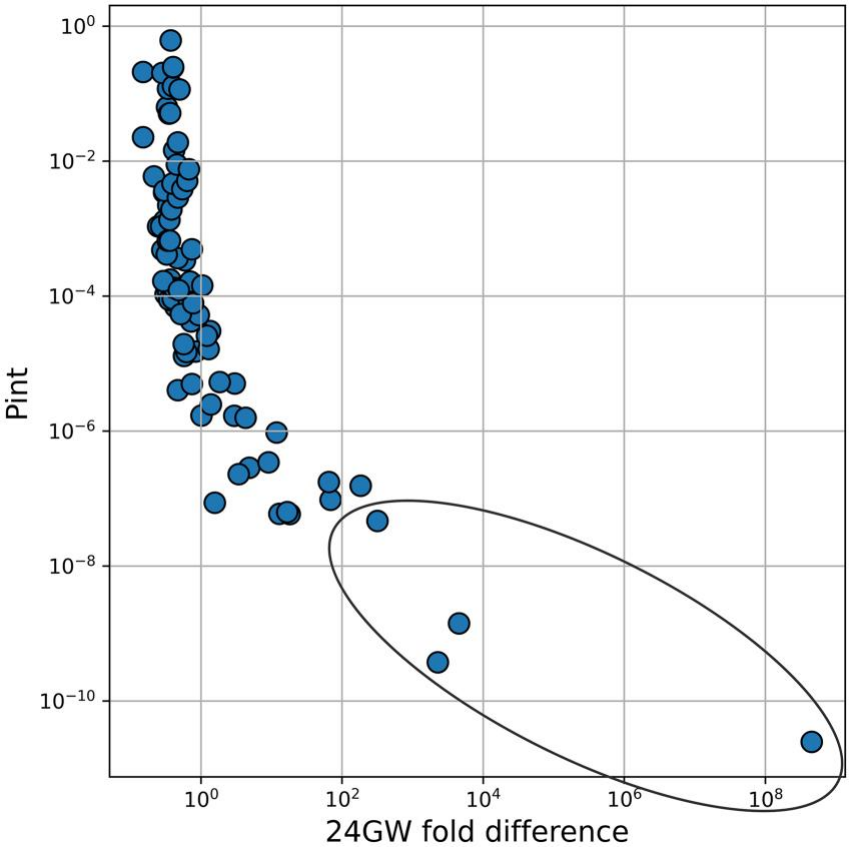
PK-Sim P_int_ distribution. PK-Sim predicted P_int_ values for all chemicals are plotted in comparison to the fold differences between C_max_ predictions from PK-Sim and the other PBPK platforms at the 24 GW timepoint. Chemicals that differed the most from the other platforms are circled.

Although P_eff_ and P_int_ are similar, the 2 values have different units and are considered differently in modeling, and thus should not be compared directly. Figure 8 illustrates the contribution of permeability to the differences seen between the PK-Sim predictions and those obtained from the other platforms. Assessment of the 4 major outliers in PK-Sim shows these to be the chemicals with the lowest P_int_ values (circled in the Fig. 8): Abamectin, cytarabine hydrochloride, methotrexate, and pravastatin. This suggests that P_int_, and how it is calculated, could be a key factor underlying the distinctly lower C_max_ values in PK-Sim.

#### Customization and accessibility

Table 6 compares the key features of the 3 PBPK modeling platforms used for this DNT-IVIVE approach. All models support a wide range of customization of model parameter inputs. GastroPlus and PK-Sim are particularly robust in this regard, offering customization options that could be useful in tailored chemical-specific approaches or follow-up assessments. The GUIs offered by GastroPlus and PK-Sim also provide a more user-friendly interface than httk, which relies on R coding. The GastroPlus environment is particularly user-friendly, as nearly all aspects of the model can be modified directly in the platform, and a thorough user manual and help are provided within the platform. The PK-Sim GUI is somewhat more complex to navigate than that of GastroPlus, but the tool allows for a high degree of customization through the Open Systems Pharmacology tool Mobi and is fully open-source. The PK-Sim user manual, however, is only available online and is less detailed than that for GastroPlus. As PK-Sim is designed for complex tailored models, it requires a greater degree of user input as compared with GastroPlus and httk. httk is perhaps the least user-friendly of the platforms for non-coders as it generally must be run in R. The ICE resource (http://ice.ntp.niehs.nih.gov) provides a GUI to access httk models (Bell et al. 2020). Although this facilitates accessibility to httk, ICE does not run the latest release of httk and does not allow for the degree of customization that is available when running httk through R. Despite lacking a GUI, httk has advantages for risk assessment, including the diverse chemical space on which it was developed, the ability to batch, the fact that it is fully open-source, and its free availability. Even more notably, the latest versions of httk provide information on applicability domain and block users from conducting modeling chemicals outside the applicability domain, though users can override this block. Information on applicability domain is not provided in GastroPlus or PK-Sim, though it is available in ADMET Predictor, which may help the user characterize the appropriateness of their chemicals for modeling with GastroPlus. Similarly, httk does not provide applicability domain information for OPERA-derived predictions, but this information is available in both the OPERA GUI and ICE. It should be noted that most chemicals will run in each of these platforms, even if they fall outside of what might be considered the platforms’ applicability domain, with metals being one exception.

**Table 6.** Comparison of PBPK modeling platforms:customization, accessibility & FAIRness.

	Criterion	GastroPlus	httk	PK-Sim
**Customization**	Ability to change parameters, e.g. incorporate experimental data	• Physiology, physicochemical properties, f_up_, Cl_int_, barrier permeability, extensive dissolution, transport	• Physiology, physicochemical properties, f_up_, Cl_int_ • Most limited	• Physiology, physicochemical properties, f_up_, Cl_int_, barrier permeability
	Additional modules	• Extensive GI model • Active transport • Enzymatic metabolism • Population simulator • Parameter sensitivity analysis module	• HTTK-Pop for population variability	• Complex GI model • In vitro distribution • Active transport • Population simulator • Parameter sensitivity analysis module
	Number of compartments	15 in standard, 22 in pregnancy	10 in standard, 20 in pregnancy	19 in standard, 28 in pregnancy
	Routes of administration	More than 10	Oral, IV, inhalation	Oral, IV; other routes can be incorporated manually in Mobi
	ADME components	f_up_, hepatic, renal and enzymatic Cl_int_, aqueous solubility, simulated gastric fluid solubility, jejunal permeability, RBP QSAR	f_up_, hepatic and renal clearance, oral bioavailability in v2.5.0	f_up_ including specific plasma binding proteins, hepatic, renal and enzymatic Cl_int_, absorption
**Accessibility/ FAIRness**	Throughput	• Batch mode integrated in GUI • Batch mode has some limits on customization	• Designed for high-throughput chemical assessments • More limited customization facilitates batching	• Batching requires coding in R • Platform designed for tailored chemical-specific model
	Applicability domain indication	Provided in ADMET Predictor, but not directly in GastroPlus	Yes; can overwrite	No
	GUI Availability	Yes	Only via ICE^a^	Yes
	Learning curve, tutorial availability	User-friendly interface; In-person and online tutorials	Coding skills required; In-person and online tutorials	Relatively intuitive interface; Tutorials available in OSP Suite, free for academics and government; in-person and online tutorials
	User manual	Extensive help manual with equations in GUI	Documentation available on GitHub	Available online
	Publicly accessible repository	Amazon Web Services environment for nonprofit use	GitHub	GitHub
	Computing resources	Laptop	Laptop	Laptop
	Open-source	No; though free to academics	Open-source	Open-source
	Software license	Flexera	Yes	GPL license
	Community registry	LinkedIn GastroPlus User Group	CRAN	GitHub: https://github.com/Open-Systems-Pharmacology
	Software citation	GastroPlus 9.8.2, Simulations Plus, Inc., Lancaster CA	Pearce, J Stat Soft, 2017; https://github.com/USEPA/CompTox-ExpoCast-httk/tree/main/models	PK-Sim version 11, part of the Open Systems Pharmacology Suite, www.opensystems-pharmacology.org
	Adheres to software quality checklist	Yes; GPX Verification Suite	Yes	Yes

a ICE does not incorporate the latest release of httk and does not support the same degree of parameterization as the R environment.

FAIR principles (findability, accessibility, interoperability, and reusability of data) are key considerations for this DNT-IVIVE approach, which is intended to support risk assessment considerations. PBPK models can be complex, and the use of open-source code and adherence to FAIR principles support greater transparency for their application in regulatory decision-making. Of the tools examined here, GastroPlus is the least aligned with these criteria. Simulations Plus requires a paid license for most GastroPlus users, although free licenses are available for academic and health authorities, and discounts are provided for government agencies and non-profits. Although Simulations Plus provides an extensive, well-documented help manual for GastroPlus, its complete source code cannot be viewed and modified. Conversely, source code is publicly available for both httk and PK-Sim. Source code for PK-Sim is less accessible than that of httk, in the sense that it might require a greater knowledge of the platform, i.e. the use of Mobi and the code base on GitHub. Code and vignettes for both tools are fully open-source and available through GitHub. Equations can generally be found in the raw C code, and help can be accessed through R. Discussion forums exist for each platform. Further accessibility considerations are summarized in Table 6.

## Discussion

In this paper, we have described a semi-high-throughput approach to translate bioactivity concentrations from in vitro DNT assays into human-relevant doses and highlight critical drivers of chemical distribution into DNT-relevant compartments. We examined differences among 3 widely used PBPK modeling platforms that impact extrapolation from in vitro DNT assay data to human-relevant concentrations and the utility of these platforms for this DNT-IVIVE approach, which is aimed at risk assessment prioritization. Each model has its own advantages and limitations with different end goals in mind. Therefore, performing a direct comparison poses a challenging task. We aimed to provide both a high-level comparison of these PBPK modeling platforms and an overview of key parameters and model features that should be considered in conducting IVIVE for DNT. In addition, we benchmarked this DNT-IVIVE approach against in vivo PODs, which demonstrated that AEDs derived from in vitro assays generally align with in vivo PODs. We hope this body of work will inform and advance application of the DNT-IVB to hazard and risk assessment characterization—the end goal of the DNT-IVB.

### Factors impacting model predictions across PBPK platforms

Physicochemical and toxicokinetic properties are known to impact chemical distribution both into the brain and across the fetoplacental barrier (Nau and Scott 1986; Pajouhesh and Lenz 2005; Eryasa et al. 2019). Our sensitivity analysis pointed to logP and the logP-related parameters, Kp and permeability, as key drivers of chemical distribution into the fetus and brain compartments. Lipophilicity is particularly important for brain accumulation due to the high fat content of this tissue (Zeliger 2013; Chandrasekaran et al. 2018; Bharal et al. 2024).

The high number of lipophilic chemicals in our test set highlighted the importance of considering how a model handles the partitioning of such chemicals. High values of logP are known to overestimate volume of distribution using Kp partitioning methods such as Rodgers and Rowland (Lucakova et al. 2008; Pearce et al. 2017). Therefore, methods such as Lucakova and the modified Schmitt, used in GastroPlus and httk, respectively, apply an adjustment factor to f_up_, based on the supposition that f_up_ is underpredicted for highly lipophilic chemicals due to a lack of lipid binding mimicked in the in vitro assay as compared with in vivo. Although both methods greatly improve estimation of the volume of distribution, Pearce et al. (2017) note that predictions for brain Kp values are still less accurate than for most other tissues, which could be due to various uncertainties. The Poulin Kp method is also commonly used with lipophilic chemicals, so it may have been a better option for modeling in PK-Sim, at least for the more lipophilic compounds in our test set (Poulin et al. 2001). Perhaps the best method for these chemicals would be that of Poulin and Haddad, which was developed for a set of high logP chemicals, though it is not available in the platforms assessed here (Poulin and Haddad 2012). Different partitioning methods are geared toward certain classes of chemicals, so performing a batched assessment brings additional uncertainties when numerous options are available.

Follow-up assessments with PK-Sim showed that each of the primary contributors identified in the sensitivity analysis—lipophilicity, Kp, and P_int_—helped to explain the discrepancies between PK-Sim’s predictions and those of the other models. Specifically, predictions for the chemicals with the lowest logP values and P_int_ values differed the most between PK-Sim and the other platforms. Our own follow-up assessments suggest that PK-Sim may underpredict permeability (mostly intestinal) for such polar chemicals (data not shown). The much broader distribution of PK-Sim brain Kp values suggests a significant difference in how these values are calculated in PK-Sim. As noted above, PK-Sim does not apply a f_up_ adjustment factor, which could have particularly significant consequences with our chemical set, which contained a relatively large number of lipophilic chemicals. This could also help to explain differences in P_int_, as P_int_ also depends on the unbound fraction of a compound. We have conducted additional analyses (data not shown) to help address the f_up_ adjustment factor and other differences between PK-Sim as compared with GastroPlus and httk. It is worth noting that Geci et al. (2024) found that converting logP to logMA improved PK-Sim predictions; thus, this may also be worth pursuing in refining our DNT-IVIVE approach with PK-Sim (Yun et al. 2014).

An additional factor that impacts chemical distribution into the brain and fetus is how tissues are limited—perfusion vs. permeability. Although this factor is related to permeability, in our study, it did not appear to have as significant a role in chemical distribution as logP, Kp, and P_int_. All tissues in the GastroPlus models and the httk pregnancy model we used were modeled as perfusion-limited, whereas all tissues in our PK-Sim models, and the httk brain compartment, were permeability-limited. In general, the permeability-limited tissues had lower predicted C_max_ values than the perfusion-limited tissues, but this varied somewhat on a chemical-by-chemical basis and is, of course, related to factors other than the rate-limiting step of tissue distribution. It is anticipated that models with a permeability-limited brain or fetus compartment would predict concentrations more accurately, as these tissues are both permeability-limited by their respective barriers. However, due to a lack of data, this cannot be verified here.

### Uncertainties

The DNT-IVIVE modeling described here is a predictive approach, with minimal data available for benchmarking, and therefore has a high degree of uncertainty. Of particular importance, especially for parameters that have a major impact on chemical distribution, is that the tool captures the appropriate chemical space. Applicability domain is recognized as a critical consideration across in silico models (OECD 2024), but many tools still fail to fully characterize their applicability domains, and the definition of this attribute remains inconsistent. Of the 3 PBPK platforms assessed here, httk is the only 1 that informs users of chemicals falling outside its applicability domain. We chose not to exclude these chemicals as the predictions for them did not differ significantly from the rest of the test set or between platforms, but we have flagged these chemicals (online supplementary material, Data 2, tabs “15GW” and “24GW”). An indication that a chemical is outside of the applicability domain is important for assessing the validity of model predictions; without this information, users might simply take the outputs at face value without conducting follow-up assessments to better understand model predictions. The chemical space of these platforms may differ, with 1 more appropriate than another; however, due to the lack of applicability domain information provided in the modeling platforms, this cannot be determined here. Applicability domain will also differ by partitioning method, so applicability domain flags could help guide users in selecting the appropriate Kp method. ADMET Predictor, which provides physicochemical and toxicokinetic predictions for GastroPlus, does flag chemicals falling outside its applicability domain, so incorporating this information into GastroPlus might be a valuable first step in evaluating the appropriateness of this platform for a particular chemical set.

Exposure is an additional uncertainty with our DNT-IVIVE approach, both due to the assumptions being made and the limited data available. Here, we modeled a single 24-h exposure, which may underestimate chronic exposure, particularly for lipophilic, low-clearance chemicals. By using C_max_, we have been as conservative as possible, while still enabling a relatively high-throughput approach. The exposure predictions are also only rough estimates, particularly for infants, as they are for reproductive-age females, and explicit lactational exposure or hand-to-mouth behavior was not considered. Exposures for reproductive-age females were selected, as these should provide a reasonable estimate of exposure for pregnancy, and no exposure estimates are available for early life stages in the SEEM models. Models considering more complex exposure scenarios are available (Brochot et al. 2019; Kayesh et al. 2025) and could be used in subsequent screening tiers. Although models to estimate lactational exposure exist, such as in PK-Sim or SimCyp (Rowland Yeo and Wesche 2023), and can be combined with PBPK models, they are not available for GastroPlus or httk. Lactational exposure could be an important route to address in follow-up assessments, particularly for lipophilic chemicals that tend to accumulate in breast milk (Lehmann et al. 2014), which were highly represented in our test set. Outputs from httk, in particular, for the infant timepoints should be interpreted with caution, as the standard httk model does not consider ontogeny, such as for clearance processes over the first years of life, or changes in the water:lipid tissue distribution. Moreover, the httk brain–adipose model manuscript has not yet been published. Both GastroPlus and PK-Sim account for ontogeny and, therefore, may provide more accurate estimates of internal exposures for the infant timepoints.

Although the in vivo PODs and fetal cord blood to maternal plasma concentration ratios aligned well with our PBPK model predictions, these in vivo data have numerous limitations. In vivo data to benchmark such a DNT-IVIVE approach are extremely limited, as data are rarely available for postnatal brain or fetal concentrations, with not even animal data available for the chemicals assessed here. Moreover, the in vivo data that were found were available for only a subset of chemicals, varied in the species and methods used to obtain them, and are simple ratios or single values. This speaks to a need to establish central repositories of in vivo data for benchmarking IVIVE approaches, particularly as in vitro methods are increasingly being envisioned and employed for risk assessment considerations. The newly compiled EFSA DNT dataset for pesticides provides a critical step in addressing this limitation (Mangas et al. 2025). Here, we have aimed to benchmark our approach to the extent possible, using the final output of such a risk assessment approach—a POD for an in vivo study, or an AED for an in vitro study—recognizing the limitations of each approach.

Apart from questions of verification, the PBPK modeling conducted here does not capture the full complexity of tissue distribution into the brain and fetus. Active transport was not considered, nor was the intricacy of the fetoplacental or blood–brain barriers, apart from organs being permeability-limited for PK-Sim and the brain for httk. Both passive barrier permeability and active transport dictate the properties of the blood–brain barrier and fetoplacental barrier. As the primary transporters present on both the blood–brain and fetoplacental barriers are efflux transporters—P-glycoprotein P (PgP) and breast cancer resistance protein (BCRP)—our approach likely overpredicts chemical distribution into the brain and fetus, making our approach relatively conservative (Ek et al. 2012). Another source of uncertainty is the bioactive concentrations that were used, as we relied on nominal concentrations to facilitate our batched assessment. Modeling in vitro distribution, such as using the Armitage model (Armitage et al. 2014), could, indeed, be incorporated in follow-up assessments.

Population variability adds yet greater uncertainty, particularly due to the limited data that exists on population variability during pregnancy or the first 6 months of life, with the first months of life well recognized as a period of hypervariability (Ginsberg et al. 2004). Each of the platforms allows for consideration of population variability to some degree, and this is an additional consideration we have begun to explore. It is worth noting that the differences we found between the 15 and 24 GW timepoints and between the 2 w and 6 m timepoints were minimal, so the exact life stages used, at least over these time windows, do not seem to impact the DNT-IVIVE approach described here.

### Comparison of PBPK modeling platform utility

Each of these tools differs in its utility, and the approach applied here should not be considered optimized for any of them. The goal of this assessment was to provide a high-level overview of model advantages and limitations and their utility for our DNT-IVIVE approach. Each of these tools was developed with a distinct context of use in mind. Although httk was developed to be employed in a high-throughput context for chemical risk assessment, GastroPlus was developed for the pharmaceutical arena for providing a detailed understanding of chemical pharmacokinetics with a particular emphasis on chemical bioavailability. PK-Sim was developed with the focus of providing refined predictions for single chemicals. Therefore, prospective users of our approach should refine it based on the chemical space of their assessment, the tool they are using, and the end goals of their analysis.

Advantages of GastroPlus include its relatively user-friendly GUI, which includes tools such as a population simulator and sensitivity analysis module, and a thorough help manual that includes information on the test sets that feed into the development of the model. Disadvantages include its lack of a blood–brain barrier and the tool being both commercial and proprietary. On the other hand, httk has the advantages of being open-source and designed for high-throughput analyses, which are required in a tiered-testing framework for risk assessment (Thomas et al. 2019). Moreover, later releases of httk provide applicability domain annotations in the model, which neither of the other modeling platforms provide. Limitations include the inability to incorporate additional complexity into the model, such as for transporter kinetics, and lack of a GUI in the standalone version. PK-Sim provides a balance between GastroPlus and httk as it can be accessed through a GUI, whereas also being open-source. PK-Sim allows for similar degrees of complexity as GastroPlus but, like httk, is limited to an R interface for batched assessments, which is required for high-throughput assessments such as ours. PK-Sim does not offer “default” options; greater user engagement is required than for the other platforms. For example, Kp methods must be selected by the user; such decisions require the user to have a high degree of experience with PBPK modeling. Unlike GastroPlus and httk, PK-Sim has no integrated physicochemical predictor, and thus users must source all physicochemical and toxicokinetic input data for their chemicals on their own.

Our assessments of model differences point to the importance of open-access tools. Without access to each of the platforms and their underlying model code, the detailed follow-up assessments we conducted would not have been possible. This is an important consideration for risk assessors who need to openly scrutinize model results. Open-source tools and a thorough understanding of each model are particularly important for DNT due to the lack of in vivo data to benchmark non-animal predictive approaches. Overall, the 3 modeling platforms provide relatively similar predictions that are concordant with in vivo data, and differences in model structure or input assumptions help to explain differences in model predictions. Through this comparison of models, we hope to have shed light on key considerations in the selection and conduct of PBPK modeling for different PBPK platforms, particularly in a risk assessment context.

### Defining the-context-of use of this DNT-IVIVE approach

**Fig. 9.**
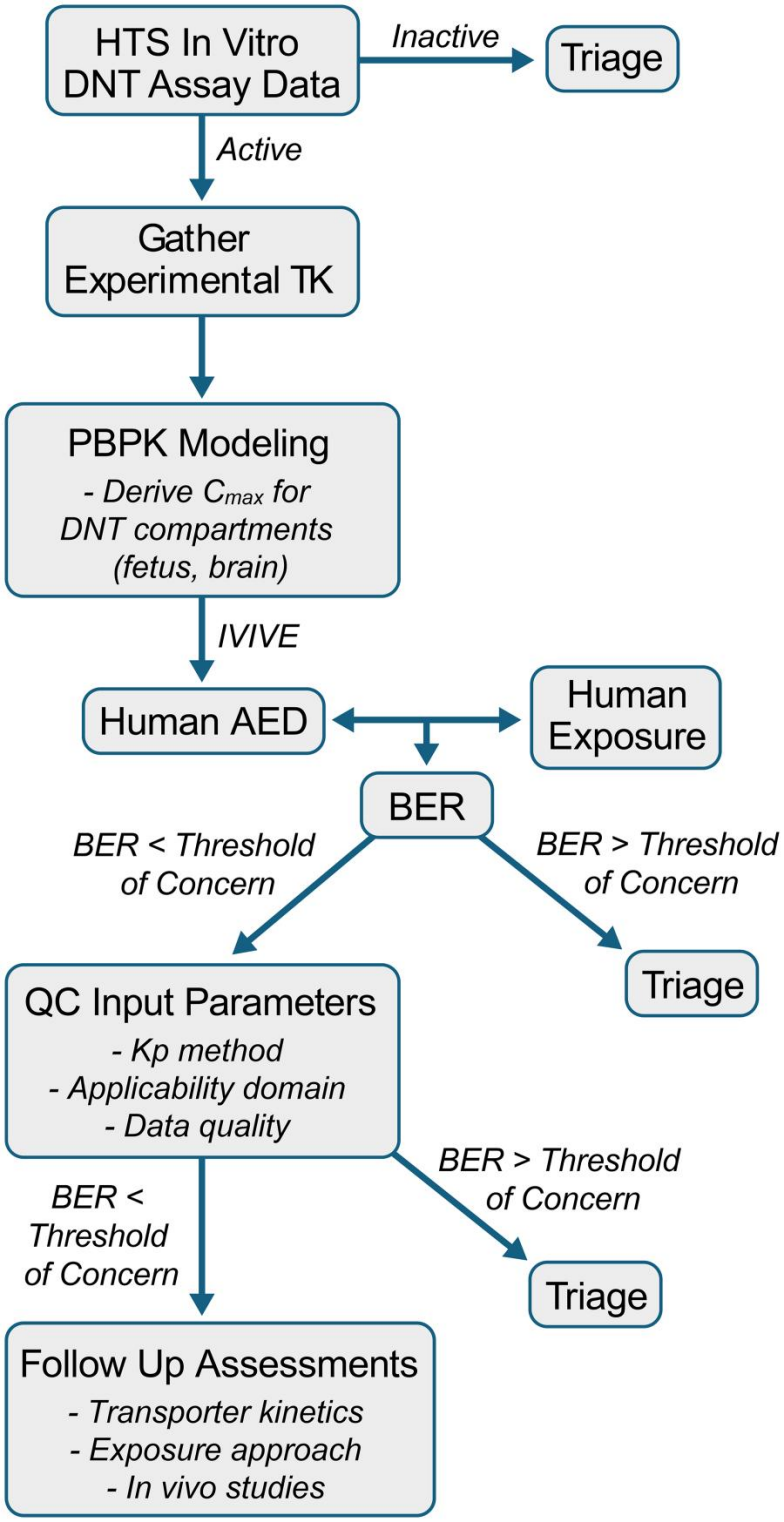
DNT-IVIVE approach for chemical prioritization for risk assessment. Following initial higher-throughput DNT-IVIVE, refinements are applied to reduce uncertainties, yielding a list of chemicals prioritized for follow-up assessments based on regulatory-defined thresholds of concern. HTS, high-throughput screening, QC, quality control; TK, toxicokinetics.

This DNT-IVIVE approach should be viewed in the context of a risk assessment prioritization framework, as we outline in Fig. 9. Due to the inputs and modeling expertise required, this approach is a middle ground between an initial high-throughput in vitro or in silico screen and a fully parameterized DNT-IVIVE approach. This prioritization framework calls for different levels of analysis depending on the purpose of the assessment and data availability. Such a triage-based approach allows for identifying chemicals likely to partition into the fetus and brain, as compared with chemicals that would likely remain in maternal plasma. Here, we have pointed out critical parameters that drive the distribution of chemicals into fetal and brain compartments. Further characterization of chemical properties that dictate chemical distribution into the brain and fetus, including for active transport processes, is needed to better inform risk for DNT.

With the many PBPK modeling and in silico physicochemical prediction tools available, our DNT-IVIVE approach provides a relatively high-throughput method for predicting distribution of chemicals into DNT-relevant compartments that requires minimal experimental data for input. Moreover, as part of this project, we developed code to allow for batching in PK-Sim. This code, which is freely accessible on Dryad and GitHub (https://doi.org/10.5061/dryad.6djh9w1fv and https://github.com/esqLABS/pregnancy-neonates-batch-run/tree/master), may help to facilitate higher-throughput modeling in PK-Sim similar to that already available for GastroPlus and httk. However, this should be applied with the understanding that while PK-Sim can be utilized for batched assessments (Geci et al. 2024), it was designed for highly curated, chemical-specific inputs and thus may be less appropriate for batched assessments than GastroPlus and httk.

Depending on the regulatory needs, chemicals prioritized in our DNT-IVIVE approach can be assessed in follow-up analyses (Fig. 9). At a minimum, a quality control review of model inputs should be performed. Such assessments would include scrutiny of raw dose–response curves, review of applicability domain limitations, and assessment of the appropriate partitioning method. If the BER or other prioritization metric still falls below a set threshold, follow-up assessments would be performed. These might include the addition of active transport to the PBPK models or the refinement of exposure. These assessments, of course, would require more data for input. Several such examples of tailored follow-up assessments exist using PK-Sim (Algharably et al. 2023; Maass et al. 2023). A tiered framework that considers in vitro data would help to increase the throughput of DNT testing for risk assessment, which has, until now, remained extremely limited. Our study also points to the potential importance of conducting follow-up assessments as needed. For example, a number of the chemicals evaluated here had a predicted C_max_ in the brain or fetus that exceeded levels in maternal plasma. In these cases, plasma concentrations, which can be readily obtained in vivo, may be a less protective measure.

In this paper, we have provided a high-throughput approach for predicting human-relevant tissue concentrations at the site and time of brain development for DNT that is relatively transferable across modeling platforms. Although we acknowledge the uncertainties of our DNT-IVIVE approach, as outlined above, the approach allows both for translation of in vitro bioactive concentrations into human-relevant doses and estimation of chemical concentrations where such measures cannot be obtained directly. Model predictions were generally consistent with available in vivo measures, though these data were extremely limited. As PODs gathered from in vivo studies are typically apical in nature, it is not possible to directly link such PODs to 1 particular in vitro DNT endpoint that may provide mechanistic information. As shown in the boxplot comparisons (Fig. 5 and online supplementary material, Document 1, Fig. 1), in vitro DNT endpoints span a wide range. For our chemical set, we found that in vivo DNT PODs fell within the range of in vitro-derived AEDs for the majority of chemicals (66%), with AEDs for most chemicals (78%) falling within what can be considered a reasonable margin of 3-fold of in vivo DNT PODs. This concordance supports both the general predictivity of these in vitro DNT assays for positive in vivo DNT and their translation into in vivo doses through our DNT-IVIVE approach. Moreover, for 72% of our chemicals, the lowest AEDs generally fell below in vivo PODs, demonstrating the potential sensitivity of this approach and its protective nature. Inclusion of the other assays in the DNT-IVB may further increase the percentage of chemicals where this holds true and is a question we are currently addressing, as noted below. Although uncertainties remain, the concordance of our model predictions with in vivo data, where available, supports the use of this approach in a tiered-testing framework for regulatory assessments for DNT.

### Ongoing activities to enhance regulatory adoption of data from the DNT-IVB

In the OECD Initial Recommendations on Evaluation of Data from the DNT In-Vitro Testing Battery (OECD 2023), various limitations in applying the DNT-IVB in regulatory adoption were identified. These included, e.g. lack of assays covering some important neurodevelopmental key processes, relative limited number of chemicals tested in the DNT-IVB, uncertainty in overall specificity and sensitivity of the DNT-IVB due to few in vivo DNT reference compounds for benchmarking, and the lack of a consensus-based strategy to be used in integrated approaches to testing and assessments (IATAs). There are ongoing activities and discussions on how to address these limitations (Cöllen et al. 2025; Debad et al. 2025). For example, the European Partnership for the Assessment of Risks from Chemicals aims to assemble a second-generation DNT-IVB by refining existing assays and develop supplementary assays covering key processes for which coverage is currently limited, e.g. microglia and astrocyte functional assays (Tal et al. 2024). The Division of Translational Toxicology at the National Institute of Environmental Health Sciences (NIEHS) has supported screening to significantly increase the number of chemicals tested in the DNT-IVB (Hall et al. in prep). Our group is currently applying a similar DNT-IVIVE approach as described here to this larger chemical set using an extended DNT-IVB. These efforts will support the use of the DNT-IVB in a weight-of-evidence assessment, but standardization of these approaches and guidance in how to make use of the DNT-IVB data in regulatory decisions are needed. The OECD, together with EFSA and the National Toxicology Program Interagency Center for the Evaluation of Alternative Methods (NICEATM), is developing an IATA framework template specific for DNT that will also provide guidance to apply quantitative IVIVE (QIVIVE). In conjunction with this, the OECD recently published a document as a supplement to the DNT-IVB guidance document (OECD 2023) that provides an overview of QIVIVE principles, PBPK modeling, and a recommended tiered approach that considers the type of model, e.g. adult, maternal, fetal, infant, to use in various contexts (OECD 2025). Additionally, at the time of submission of this manuscript, EFSA had launched a public consultation on a scientific opinion document on applying PBPK and QIVIVE using the DNT-IVB that identified various uncertainty domains (https://connect.efsa.europa.eu/RM/s/consultations/publicconsultation2/a0lTk000004vWya/pc1517). Although these are useful resources to enhance the regulatory adoption of DNT-IVB data, neither provides guidance on which model to select nor how the DNT-IVB can be realized in a high-throughput approach. Moreover, the EFSA public consultation noted the lack of comparison of IVIVE approaches for DNT to PODs from in vivo DNT studies. These points underscore the importance of the body of work presented here.

## Conclusion

We have provided a high-throughput approach for translating in vitro DNT assay data into human-relevant doses. The work described here highlights critical drivers of chemical distribution into DNT-relevant compartments and identifies model differences and limitations, while emphasizing the importance of understanding the chemical space of the modeling platforms utilized. Benchmarking of the models against the limited in vivo data available showed that our DNT-IVIVE approach is both predictive and protective. Following initial higher-throughput screening as described here, model predictions can be refined by the incorporation of more experimental data, such as on permeability, and we are currently exploring how the incorporation of such data might improve the approach. It is worth noting that a similar IVIVE approach could be applied to other developmental and reproductive toxicity endpoints using the bioactivity data and corresponding tissue concentrations for each endpoint. IVIVE for other target tissues would likely differ in their applicability domain considerations. We suggest employing such a DNT-IVIVE approach as outlined here to facilitate interpretation and use of in vitro approaches in risk assessment for DNT. Translation of in vitro DNT bioactivity into human-relevant doses to facilitate the interpretation and use of in vitro approaches for risk assessment for DNT would meet a critical need in this area due to the lack of existing data on the DNT potential of chemicals and the increasing rates of diagnoses for neurodevelopmental disorders.

## Supplementary Material

kfaf147_Supplementary_Data

## Data Availability

Data are available at https://doi.org/10.5061/dryad.6djh9w1fv
