# Supplementary material for: Comparison of physiologically based pharmacokinetic modeling platforms for developmental neurotoxicity in vitro to in vivo extrapolation
Source: Toxicol Sci. 2025 Oct 27;209(2):kfaf147. doi: 10.1093/toxsci/kfaf147 (PMC13397022; doi:10.1093/toxsci/kfaf147)
Supplement: kfaf147_Supplementary_Data [file kfaf147_supplementary_data.zip › toxsci-25-0428-File014.docx]

**Supplementary Table 1. Distribution of C_max_ values derived from PBPK modeling simulations for the three platforms and four life stages assessed. GP: GastroPlus; PK: PK-Sim.**

|  | **C_max_ Distribution by PBPK model, Compartment, and Life-Stage** | | | | | | | | | | | |
| --- | --- | --- | --- | --- | --- | --- | --- | --- | --- | --- | --- | --- |
|  | **Med (Min-Max) C_max_ (µM)** | | | | | | | | | | | |
|  | **Pregnancy** | | | | | | **Pediatric** | | | | | |
| **Compartment** | **GP**  **15GW** | **httk**  **15GW** | **PK**  **15GW** | **GP 24GW** | **httk 24GW** | **PK**  **24GW** | **GP 2w** | **httk 2w** | **PK 2w** | **GP 6m** | **httk 6m** | **PK 6m** |
| **Plasma** | 1.561 | 0.838 | 0.393 | 1.488 | 0.786 | 0.35 | 1.475 | 1.499 | 0.338 | 1.688 | 1.591 | 0.323 |
|  | 0.059-33.25 | 0.003-7.576 | 1E-4-13.44 | 0.057-31.65 | 0.003-6.993 | 5E-5-13.57 | 0.007-25.51 | 0.063-14.35 | 6E-5-12.25 | 0.013-27.498 | 0.061-14.32 | 2E-5-12.09 |
| **Fetal Venous** | 1.325 | 0.524 | 0.458 | 1.227 | 0.46 | 0.188 |  |  |  |  |  |  |
|  | 0.037-10.04 | 0.003-4.845 | 1E-8-5.872 | 0.031-15.73 | 0.002-4.229 | 4E-9-3.317 |  |  |  |  |  |  |
| **Fetus** | 0.655 | 2.014 | 0.708 | 1.518 | 1.798 | 2.741 |  |  |  |  |  |  |
|  | 0.037-3.536 | 0.007-7.365 | 8.49E-9-3.034 | 0.073-3.676 | 0.006-5.879 | 2E-9-6.699 |  |  |  |  |  |  |
| **Brain** |  | 1.101 |  |  | 0.997 |  | 2.309 | 2.161 | 0.875 | 3.524 | 2.202 | 0.77 |
|  |  | 0.005-5.688 |  |  | 0.004-5.322 |  | 0.016-9.857 | 0.016-6.008 | 2E-8-5.593 | 0.028-12.53 | 0.013-5.959 | 7E-9-5.832 |

**
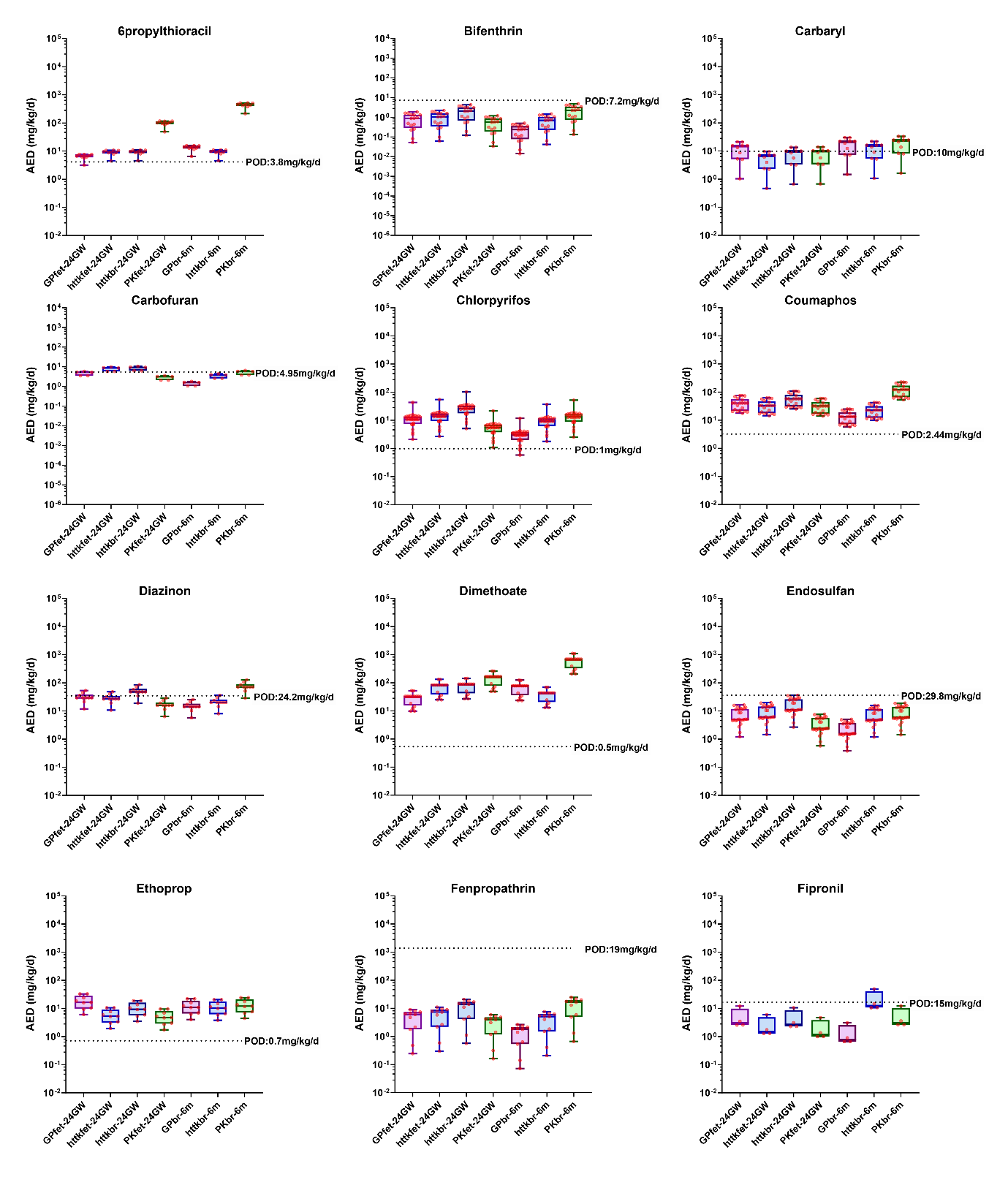

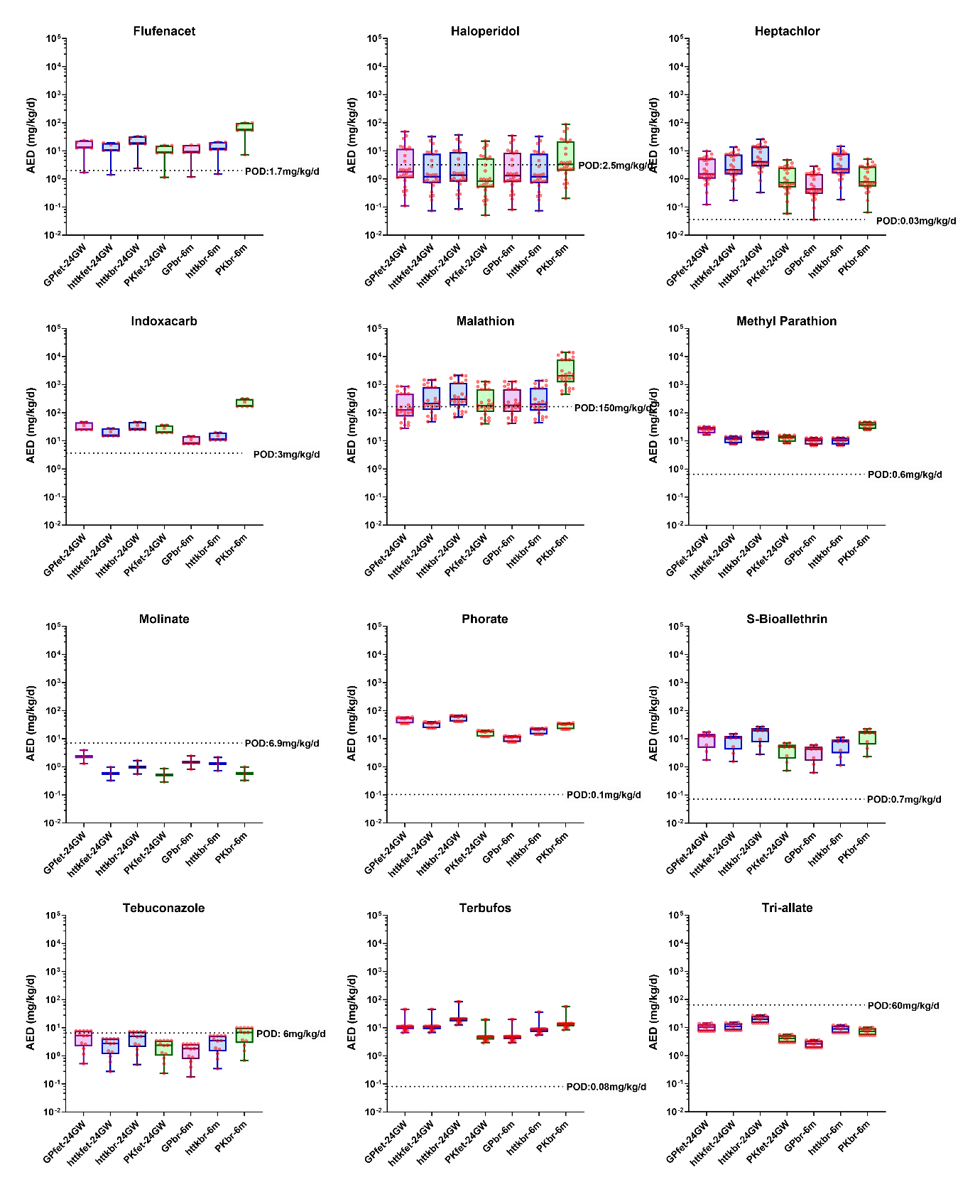

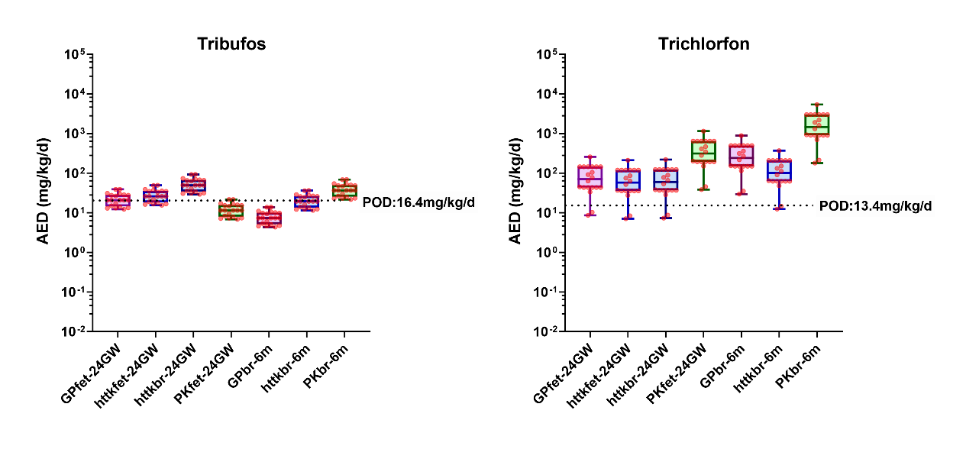
**

**Supplementary Figure 1.** Comparison of in vitro DNT-IVIVE-derived AEDs against curated in vivo DNT PODs. AEDs for each of the bioactive endpoints are plotted in red for the three platforms at 24GW and 6m. For comparative purposes, the y axis is scaled to the same degree for all chemicals.
